# Phytochemical and antioxidant analysis of medicinal and food plants towards bioactive food and pharmaceutical resources

**DOI:** 10.1038/s41598-021-89437-4

**Published:** 2021-05-11

**Authors:** Manyou Yu, Irene Gouvinhas, João Rocha, Ana I. R. N. A. Barros

**Affiliations:** 1 Centre for the Research and Technology of Agro-Environmental and Biological Sciences, CITAB, University de Trás-os-Montes e Alto Douro, UTAD, 5000-801 Vila Real, Portugal; 2 Chemistry Centre-Vila Real, CQ-VR, UTAD, 5000-801 Vila Real, Portugal; 3 Department of Chemistry, School of Life Sciences and Environment, UTAD, Quinta de Prados, 5001-801 Vila Real, Portugal

**Keywords:** Biochemistry, Chemistry

## Abstract

Plants with medicinal properties play an increasingly important role in food and pharmaceutical industries for their functions on disease prevention and treatment. This study characterizes the phenolic composition and antioxidant activity of seven medicinal and food plants, including the leaves of *Salvia officinalis* L., *Rosmarinus officinalis* L., *Olea europaea* L., and *Punica granatum* L., as well as the leaves and young stems of *Ruta graveolens* L., *Mentha piperita* L., and *Petroselinum crispum*, Mill., by using colorimetric, chromatographic, and spectrophotometric assays. Results revealed that the hydro-methanolic leaf extracts of *P. granatum* (pomegranate) displayed the highest content of total phenols (199.26 mg gallic acid per gram of plant dry weight), *ortho*-diphenols (391.76 mg gallic acid per gram of plant dry weight), and tannins (99.20 mg epicatechin per gram of plant dry weight), besides a higher content of flavonoids (24 mg catechin per gram of plant dry weight). The highest antioxidant capacity measured by ABTS, DPPH, and FRAP (2.14, 2.27, and 2.33 mM Trolox per gram of plant dry weight, respectively) methods was also obtained in pomegranate leaf extracts, being 4–200 times higher than the other species. Such potent antioxidant activity of pomegranate leaves can be ascribed to the presence of different types of phenolic compounds and the high content in tannins, whilst phenolic acids and flavonoids were found to be the dominant phenolic classes of the other six plants. Consequently, despite the well-known antioxidant properties of these plant species, our study suggests pomegranate leaf can stand out as a relatively more valuable plant source of natural bioactive molecules for developing novel functional food-pharma ingredients, with potential for not only promoting human health but also improving bio-valorization and environment.

## Introduction

The recent development of functional foods and pharmaceutical products based on medicinal and food (namely fruits and vegetables) plants has brought improvements to all aspects of life, including the alleviation of physical disorders, the reduction in the use of synthetic antibiotics, and the increase in life expectancy^1,2^. Indeed, these plants have long been used as safe, effective and sustainable sources of natural antioxidants or free radical scavengers, particularly phenolic compounds, such as phenolic acids, flavonoids, tannins, stilbenes, and anthocyanins^2^. Those phenolics are mostly regarded to confer upon the antioxidant activity of medicinal and food plants, making a marked contribution in the fight against many pathological conditions such as cancer, diabetes, aging, cardiovascular, and other degenerative diseases^2–5^.

*Salvia officinalis* L., *Rosmarinus officinalis* L., and *Mentha piperita* L. commonly named as sage, rosemary, and peppermint, respectively, belongs to the family of Lamiaceae. They are well-known herbs and spices used in foods for flavors and aromas. Infusions, leaves or essential oils of its each species are reported to possess therapeutics in anti-cancer, anti-microbial, anti-diabetes, and gastrointestinal diseases, etc.^3, 6–8^. Several bioactivities of sage like antinociceptive, hypolipidemic, and memory-enhancing effects have been demonstrated with clinical trials^7^. Rosmarinic acid is abundant both in sage and rosemary, contributing to their anti-inflammatory properties^3, 6, 7^. Flavonoids, phenolic lignans and stilbenes, and essential oils are expected to be responsible for the aroma effects of peppermint^8^.

Rue (*Ruta graveolens* L.) has been one of the key plants of the European pharmacopoeia since ancient times for the use in tremors, paralysis, nervine disorders, and joint pain^9^. And nowadays, it becomes medicine in Mediterranean region, due to its prominent biological activities, especially neuroprotection^9,10^. Rutin, psoralen, limonene, and pinene are reported as main constituents in this plant extracts or rue oils^9,10^.

Olive (*Olea europaea* L.) oil is one of the major components of the Mediterranean diets. Recently, phenolics present in olive leaves, especially the oleuropein, are reviewed to be potential economic and renewable source of natural by-products, attributed to its antioxidant, antihypertensive, hypoglycemic, hypocholesterolemic and cardioprotective activity^11,12^.

Parsley (*Petroselinum crispum* Mill.), used as culinary and medicinal herb, is originated from Mediterranean region. Phytochemicals particularly apigenin, coumarins, myristicin, and apiol are active compounds rich in parsley leaves, exhibiting diverse pharmacological properties, such as cyto-, gastro-, brain-, nephron-protective effects, and so on^13–15^.

Pomegranate (*Punica granutum* L.) a deciduous shrub in the family of Lythraceae, is one of the oldest known plants. Both the edible (namely fruit juice) and non-edible parts (including seeds, peels, leaves, roots and bark) of this plant have been evidenced to have a wide range of health benefits, largely resulting from its abundant phenolic acid, flavonoids, tannins, amino acids, and alkaloids^16,17^. However, the importance of pomegranate leaves, as agricultural and industrial waste, is of great interest and value to be emphasized by means of describing its beneficial effects and studies performed on this field.

Within the frame, materials from the seven medicinal and food plants aforementioned, that is, leaves and young stems (easy for picking) of rue, peppermint, and parsley, as well as the leaves of sage, rosemary, olive, and pomegranate are outstanding for their higher levels of phenolic contents and antioxidant capacities, along with relatively lower (dose-dependent) or inexistent toxicity^6–9,11,13,15,17^. Therefore, in an attempt to explore plant-based alternative solutions in promoting health, as well as paving the way towards our future pre-clinical and clinical studies, we aimed to analyze the phenolic classes (total phenols, *ortho*-diphenols, flavonoids, and tannins) and antioxidant activities of different plant species under the same evaluation condition. Furthermore, the principal phenolic constituents were chromatographically characterized to investigate the relationship between the phenolic content and antioxidant activity.

## Results and discussion

### Phenolic content of tested medicinal and food plants

Results of colorimetric and spectrophotometric analysis of seven medicinal and food plants were showed in Table 1. In general, the total phenolic content of the selected plant species was found to be at the highest level in pomegranate leaf extracts at 199.26 mg of gallic acid equivalents per gram of plant dry weight (mg GAE g^−1^ DW), followed by three *Lamiaceae* species, including peppermint (70.06 mg GAE g^−1^ DW), sage (50.89 mg GAE g^−1^ DW) and rosemary (48.48 mg GAE g^−1^ DW). On the contrary, parsley displayed the lowest value of total phenols (6.94 mg GAE g^−1^ DW). The same trend was observed concerning the content of *ortho*-diphenols and tannins of all investigated samples, reporting the following sequence: pomegranate > peppermint > sage > rosemary > rue > olive > parsley. The *ortho*-diphenol and tannin content of the methanolic extracts ranged from 26.40 to 391.76 mg GAE g^−1^ DW, and from 1.33 to 99.20 mg of epicatechin equivalents per gram of plant dry weight (mg ECE g^−1^ DW), respectively. Moreover, results on total flavonoids content showed a different pattern compared to other phenolic classes, with peppermint showing maximum values at 70.14 mg of catechin equivalents per gram of plant dry weight (mg CATE g^−1^ DW), following with rosemary (49.14 mg CATE g^−1^), sage (43.92 mg CATE g^−1^), and pomegranate (24.34 mg CATE g^−1^). Furthermore, the flavonoid content of olive leaf was higher than that of rue, in contrast to the trend of the other phenolic classes. Rosemary and sage had comparatively high levels of flavonoids, while the minimum values were reported for parsley.

**Table 1:** Phenolic content and antioxidant activity of hydro-methanolic extracts of the studied medicinal and food plants.

Medicinal/food plant	Total phenols (mg GAE g^−1^ DW)	*Ortho*-diphenols (mg GAE g^−1^ DW)	Flavonoids (mg CATE g^−1^ DW)	Tannins (mg ECE g^−1^ DW)	ABTS (mM Trolox g^−1^ DW)	DPPH (mM Trolox g^−1^ DW)	FRAP (mM Trolox g^−1^ DW)
*Salvia officinalis* L.	50.89 ± 0.37^c 1^	169.68 ± 1.41^c^	43.92 ± 0.05^c^	28.02 ± 1.40^c^	0.27 ± 0.01^c^	0.25 ± 0.00^c^	0.40 ± 0.00^c^
*Rosmarinus officinalis* L.	48.48 ± 0.13^d^	133.88 ± 1.78^d^	49.14 ± 0.83^b^	18.10 ± 0.77^bc^	0.27 ± 0.01^c^	0.26 ± 0.01^c^	0.42 ± 0.01^c^
*Ruta graveolens* L.	24.96 ± 0.19^e^	100.52 ± 0.54^e^	11.90 ± 0.10^f^	9.60 ± 0.33^ab^	0.10 ± 0.00^d^	0.12 ± 0.00^d^	0.16 ± 0.00^d^
*Olea europaea* L.	23.52 ± 0.30^e^	58.74 ± 0.81^f^	16.96 ± 0.38^e^	7.09 ± 0.63^a^	0.11 ± 0.00^d^	0.11 ± 0.00^d^	0.15 ± 0.00^d^
*Mentha piperita* L.	70.06 ± 1.01^b^	179.31 ± 2.04^b^	**70.14 ± 1.23**^**a**^	69.91 ± 2.80^d^	0.35 ± 0.02^b^	0.38 ± 0.01^b^	0.50 ± 0.01^b^
*Petroselinum crispum* Mill.	**6.94 ± 0.32**^**f**^^**2**^	**26.40 ± 0.32g**	**0.76 ± 0.09**^**g**^	**1.33 ± 0.08**^**a**^	**0.01 ± 0.00**^**e**^	**0.01 ± 0.00**^**e**^	**0.01 ± 0.00**^**e**^
*Punica granatum* L.	**199.26 ± 1.16**^**a**^	**391.76 ± 1.10**^**a**^	24.34 ± 0.98^d^	**99.20 ± 2.30**^**e**^	**2.14 ± 0.04**^**a**^	**2.27 ± 0.02**^**a**^	**2.33 ± 0.04**^**a**^
*p* value	**	**	**	**	**	**	**

^1^Values are presented as mean ± SD (n = 3) for each phenolic group. Mean values followed by different superscript lowercase letters report significant differences between different plant extracts at **p < 0.001, according to Tukey’ s multiple range test.

^2^Values in bold represent the highest and the lowest for each parameter assessed.

Different phenolic contents of different plant samples have been reported in the literature^12,18–25^. For instance, the total phenol content of sage and peppermint was 27.94 and 45.25 mg GAE 100 g^−1^ DW, meanwhile the flavonoid content of them was 27.54 and 25.17 mg catechin per 100 g, which were much lower than that of our results^19^. Parsley extracts had 1.583 GAE mL^−1^ of total phenols, 0.091 mg catechin mL^−1^ of flavonoids, and 1.167 mg catechin mL^−1^ of condensed tannins^26^. Salama et al. ^12^ described significant differences in the amounts of total phenolics, flavonoids, and tannins of olive leaves, under different extraction solvents, ranging from 42.02 to 85.50 mg GAE g^−1^, 31.22 to 105.19 mg quercetin g^−1^, and 30.92 to 51.03 mg tannic acid g^−1^, respectively. The contents of phenolic and flavonoid compounds in rue were 14.1 GAE g^−1^ and 15.8 mg rutin g^−1^ of dry extracts^20^. Some studies^27–29^ have evidenced considerably high level of phenolics in pomegranate leaf extracts, up to 328 mg GAE g^−1^ DW. Interestingly, pomegranate leaves are characterized by carbohydrates, reducing sugars, sterols, saponins, flavonoids, ellagitannins, piperidine alkaloids, flavones, glycosidic compounds, which are the richest source of phytochemicals when considering the non-edible parts of this species, some food products (red wine, green tea, etc.), and another 109 medicinal plants^30–32^. Our results disclosed that tannins were the main phenolic compounds of pomegranate leaf extract, which has also been corroborated by other studies^33^.

As shown in data (Table 1), significant differences (*p* < 0.001) around 29, 15, 92 and 75 times were observable respectively for total phenols, *ortho*-diphenols, flavonoids and tannins in the seven plant extracts, indicating that each phenolic classes exhibited considerably different content among the studied plants. This result was in agreement with other authors^34^, who found that depending on the plant species and botanical family, strong differences were found among 10 medicinal herbs and 11 spices. Meanwhile, the same authors^34^ observed a wide variance of phenolics in different samples of the same species, such as the total phenolic content of nine independent samples of peppermint was from 18.3 to 284.3 mg GAE g^−1^. Moreover, contents of total phenolics, flavonoids, and condensed tannins of 13 different provenances of rosemary, collected in different seasons ranged from 22.46 to 44.57 mg GAE g^−1^ DW, from 1.49 to 5.01 mg quercetin g^−1^, and from 0.81 to 1.71 mg CATE g^−1^ DW, respectively^18^. Our results showed inconsistency with this observation, probably attributed to the varieties, or geographical differences, as well as to the collection time, agroclimatic conditions and other relevant factors^24,25^. However, to some extent, pomegranate leaf was supposed to have a relatively higher phenolic content than many other medicinal plants. Therefore, it can be inferred that pomegranate leaf could be an important valuable source of bioactive compounds for medicinal purposes and health care.

In addition, in the current study, the colorimetric analysis of flavonoids varied between pomegranate leaf (orange-yellowish) with other plants (pink) and the standard (catechin, pink) under the same conditions (as below described in the methods). This visual observation may be related to the fact that leaves from pomegranate have different predominant sub-classes of flavonoids, different from that existing in the other studied plants^32^. So, the methodology, especially to normalize the use of standards such as quercetin or rutin^35^ should be modified to accurately quantify the amount of flavonoids.

### In vitro antioxidant activity

The in vitro antioxidant activity assays were carried out to assess the capacity of plant extracts to scavenge free radicals including 2,2′‐azino‐bis(3‐ethylbenzothiazoline‐6‐sulfonic acid radical cation (ABTS^+·^) and 2,2‐di(4‐tert‐octylphenyl)‐1‐picrylhydrazyl radical (DPPH·), as well as the ability to reduce ferric (III) iron to ferrous (II) iron. Overall, Table 1 revealed that all the species displayed high antioxidant capacities, although significant differences were observed (*p* < 0.001), ranging from 0.01 to 2.14 mM Trolox per gram of plant dry weight (mM Trolox g^−1^) for ABTS, from 0.01 to 2.27 mM Trolox g^−1^ for DPPH, and from 0.01 to 2.33 mM Trolox g^−1^ for FRAP (ferric reducing antioxidant power), with large variation over 210-fold. It was found that pomegranate always exhibited the highest antioxidant properties (2.14–2.33 mM Trolox g^−1^) throughout the three measurements, followed by peppermint (0.35–0.50 mM Trolox g^−1^), sage (0.27–0.40 mM Trolox g^−1^), rosemary (0.27–0.42 mM Trolox g^−1^), rue (0.10–0.16 mM Trolox g^−1^), and olive leaf (0.11–0.15 mM Trolox g^−1^). No significant difference was observed between sage and rosemary, and between rue and olive leaf. However, parsley extracts reported the lowest antioxidant potential (0.01 mM Trolox g^−1^).

Previous data regarding the antioxidant capacities of sage, rosemary, rue, olive leaf, peppermint, parsley, and pomegranate leaf have been reported by several authors^12,14,18,22,26,31,36^. The IC_50_ values of ABTS and DPPH radical scavenging activity, as well as the EC_50_ values of reducing powder regarding olive leaves ranged from 20.13 to 190.95 µg mL^−1^, from 17.97 to 41.64 µg mL^−1^, and from 90 to 216 µg mL^−1^, arising from diverse extraction solvents ^12^. Rosemary leaves displayed 75.04 and 9.08 µg mL^−1^ of IC_50_ by ABTS and DPPH assay, along with 4.12 µM by FRAP method ^18^. Farnad et al. ^22^ reported the methanol-ethanol (1:1) extract of peppermint had the best DPPH radical scavenging ability (10.05 mg mL^−1^ of IC_50_) and ferric reducing power (184.22 µmol per 100 g powder). The ethanolic extract of parsley displayed 0.34 mg AAE mL^−1^ (milligrams of ascorbic acid equivalents per milliliter) of DPPH and 0.942 mg AAE mL^−1^ of FRAP, which was correlated with the anti-glycation activity of this extract^26^. The best antioxidant capacities conducted by DPPH (17.09% of IC_50_) and FRAP (458.26 mmol Fe^II^ L^−1^) were determined for sage leaves which were collected in May ^36^. Cefali et al. ^14^ stated the rue extracts exhibited antioxidant potential against DPPH (281.02 µg mL^−1^ of IC_50_) and ABTS (587.98 µg mL^−1^ of IC_50_) radicals, indicating the premature aging protective effect.

Importantly, several studies in vitro and in vivo have recorded the superior antioxidant capacity of pomegranate leaves by contrast with its non-edible parts, of which leaves are as effective as peels in the anti-bacterial, analgesic, acute and chronic anti-inflammatory effects^37,38^, while more potent than flowers, stems, and seeds^31,39–42^. Authors proved the potency of pomegranate leaf was higher than that of flower in the prevention of ethylene glycol-induced nephrolithiasis, in the inhibition of DPPH and hydroxyl radicals, and in the reduction of ferric iron^39,40^. Data^41^ highlighted leaves worked more effectively than stems and led to the most loss of MMP (mitochondrial membrane permeability) potential, consequently suggested as an anti-cancer and anti-proliferative agent. Elfalleh et al. ^31^ illustrated the highest reducing power (348.68 µg mL^−1^ of EC_50_) occurring in the aqueous extract of pomegranate leaf. Furthermore, a higher antioxidant and enzyme inhibitory activity was exposed in two extracts (methanolic and water) of pomegranate leaves among different fruit tree leaves^28^. The ethanolic extracts of pomegranate leaf also exhibited remarkable antioxidant and anti-glycation ability of twenty edible and medicinal plants^29^. The level of anti-radical and ferric reducing properties of pomegranate leaves in our results was similar to some authors^42^. However, comprehensively comparative research involving in the phytochemical and antioxidant properties between pomegranate leaves and other numerous medicinal plants is still scarce; Widely practical application of pomegranate leaf hasn’t come into being, although different biological activities of this material extracts are studied increasingly. Many authors have deeply reviewed for sage, rosemary, peppermint, rue, parsley, and olive leaf. Thus it is worth stressing on the brilliant phenolics and antioxidant property of pomegranate leaves, and developing high added-value products from these materials in the food, pharmaceutical, or even nutraceutical and cosmeceutical industries.

### Chromatographic analysis of phenolic compounds

With the development of chromatographic techniques, the phenolic chemistry of many plants has been explored and analyzed to a certain degree, providing us important reference data. To obtain a more complete picture of the quality and quantity of phenolic constituents in the selected plants, 64 phenolic compounds were identified (Table 2), of which 59 were quantified with authentic standards relying on RP-HPLC-DAD, as well as by comparison with the literature (retention time, UV/Visible λ_max_, and spectra). Concentrations of identified phenolics were expressed as milligram per gram dry weight of plant (mg g^−1^).

**Table 2:** Phenolic profiles of the studied plants and concentrations of their identified compounds (mg g^−1^ DW of plants).

Tentative identification	RT (min)	HPLC–DAD λ _max_ (nm)	Sage leaf	Rosemary leaf	Rue leaf and stem	Olive leaf	Peppermint leaf and stem	Parsley leaf and stem	Pomegranate leaf	LSD ^2^ (*p* < 0.05)	*p* value
**Phenolic acids (derivatives)**											
2,3-Hydroxybenzoic acid	11.71	231, 251, 324	0.03^b^^1^	–^3^	–	0.02^b^	–	0.40^a^	–	0.27	**
Ellagic acid derivative I	13.15	256, 357	–	–	–	–	–	–	1.75	–	–
Gallic acid	14.45	232, 277	–	–	–	0.16	–	–	–	–	–
Gallic acid derivative I	14.57	231, 279	0.19^b^	0.09^c^	0.25^a^	–	–	0.25a	–	0.08	**
Neochlorogenic acid	16.39	233, 299, 333	–	–	1.96a	0.25c	0.46b	0.14d	–	1.14	**
Galloy glucose	17.23	232, 272	–	–	–	–	–	–	5.30	–	–
Coumaric acid	18.28	234, 311, 378	–	–	0.84	–	–	–	–	–	–
Chlorogenic acid	18.66	233, 326, 378	–	–	1.54^a^	0.26^b^	0.28^b^	–	–	0.57	**
Caffeic acid	19.97	233, 325	0.79^a^	0.17^c^	–	–	0.35^b^	–	–	0.11	**
Vanillic acid	20.36	233, 274, 335	0.79^a^	–	0.57^b^	0.03^c^	–	–	–	0.11	**
Ellagic acid derivative II (rhamnoside)	21.34	234, 275, 378	–	–	–	–	–	–	4.58	–	–
Ellagic acid	24.30	254, 369	–	–	–	–	–	–	4.60	–	–
Rosmarinic acid	27.48	237, 290, 330	**4.61**^a4^	**4.31**^b^	–	–	0.23^c^	–	–	0.68	**
Rosmanol	36.08	234, 277, 333	0.22^b^	0.35^a^	–	–	–	–	–	–	**
Epirosmanol	37.09	234, 288, 318	0.05^b^	0.26^a^	–	–	–	–	–	–	*
Carnosol	40.81	235, 288, 324	0.24^a^	0.05^b^	–	–	–	–	–	–	*
Carnosic acid	41.09	236, 289, 325	0.15^b^	0.50^a^	–	–	–	–	–	–	**
**Flavonoids (derivatives)**											
Gallocatechin	2.39	234, 265	2.10^b^	0.74^e^	1.67^c^	0.72^e^	1.46^d^	2.30^a^	–	0.76	**
Catechin	18.31	233, 284	1.89c	3.61a	–	0.77d	0.43e	2.53b	–	0.47	**
Myricitin-3-*O*-glucoside	20.01	232, 252, 354	–	–	–	–	–	0.30	–	–	–
Luteolin glycoside I	22.40	233, 253, 266, 348	–	–	–	–	1.38	–	–	–	–
Epicatechin	22.59	232, 279	–	–	–	–	–	**3.72**	–	–	–
Eriodictyol glycoside I (rutinoside)	23.46	235, 280, 327	–	–	–	–	**20.33**	–	–	–	–
Rutin (quercetin-3-*O*-rutinoside)	23.85	233, 253, 265, 354	–	–	**26.10**^a^	0.86^c^	**9.90**^b^	–	–	0.77	**
Luteolin glycoside II (rutinoside)	23.87	233, 255, 265, 349	–	–	–	–	3.10	–	–	–	–
Luteolin-7-*O*-glucoside	24.63	233, 253, 265, 349	–	–	–	0.62	–	–	–	–	–
Luteolin glycoside III	25.17	233, 253, 272, 345	–	1.52	–	–	–	–	–	–	–
Eriodictyol glycoside II	25.29	233, 284, 325	–	–	–	–	0.81	–	–	–	–
Quercetin glycoside I	25.44	233, 254, 355	–	–	1.13	–	–	–	–	–	–
Apigenin glycoside I	25.44	233, 266, 337	–	–	–	0.16	–	–	–	–	–
Luteolin glycoside IV (glucuronide)	25.45	233, 255, 266, 347	–	–	–	–	1.36	–	–	–	–
Luteolin glycoside V	25.51	233, 266, 346	3.42^a^	0.45^b^	–	–	–	–	–	–	**
Apigenin-7-*O*-apiosylglucoside	25.74	237, 266, 335	–	–	–	–	–	**4.04**	–	–	–
Diosmetin glycoside	25.99	233, 266, 348	–	–	–	–	–	0.09^b^	0.39^a^	–	*
Eriodictyol-7-*O*-rutinoside	26.07	233, 284, 327	–	1.71^b^	–	–	**8.17**^a^	–	–	–	**
Diosmetin glycoside isomer	26.28	234, 267, 341	–	–	–	–	–	1.08	–	–	–
Apigenin glycoside II (glucoside)	26.45	234, 268, 333	–	–	–	0.48^b^	–	–	2.93^a^	–	*
Luteolin glycoside VI (glucoside)	27.16	234, 268, 341	–	–	–	–	–	–	1.25	–	–
Apigenin glycoside III (rutinoside)	27.22	237, 266, 337	**6.58**^a^	0.11^b^	–	–	–	–	–	–	**
Apigenin glycoside IV	27.81	234, 286, 337	0.37^b^	–	–	–	0.67^a^	0.17^c^	–	0.27	**
Luteolin-3-*O*-glucuronide	28.17	235, 268, 341	–	1.99	–	–	–	–	–	–	–
Luteolin glycoside VII	28.41	234, 268, 341	–	–	–	–	–	–	0.65	–	–
Luteolin	30.40	234, 288, 348	–	–	–	0.79^a^	0.15^b^	–	–	–	*
Quercetin	30.72	233, 283	–	–	–	0.22^b^	–	0.51^a^	–	–	*
Naringenin	32.21	233, 282	–	–	–	–	–	I^5^	–	–	–
Hesperidin	32.41	234, 289	–	–	–	–	–	I	–	–	–
Epicatechin gallate	37.66	234, 274, 401	–	–	**7.82**^a^	1.33^b^	1.27^c^	0.19^d^	–	1.81	**
Apigenin	40.37	236, 333	–	–	0.93	–	–	–	–	–	–
**Tannins**											
Punicalin	12.48	231, 274	–	–	–	–	–	–	1.32	–	–
Ellagitannin I (Castalagin derivative)	18.96	234, 268, 378	–	–	–	–	–	–	**56.06**	–	–
Granatin B	20.34	233, 267, 378	–	–	–	–	–	–	1.99	–	–
Ellagitannin II	22.54	238, 273	–	–	–	–	–	–	**45.16**	–	–
Ellagitannin III	23.31	234, 274	–	–	–	–	–	–	4.86	–	–
Ellagitannin IV	24.59	234, 275	–	–	–	–	–	–	4.24	–	–
Ellagitannin V	25.27	232, 279	–	–	–	–	–	–	2.04	–	–
**Phenylethanoids**											
Tyrosol	17.82	231, 264, 330	–	**4.56**^a^	0.19^c^	0.75b	–	–	–	2.42	*
Verbascoside	23.26	233, 288, 299, 330	–	–	–	0.26	–	–	–	–	–
Oleuropein derivative I	26.84	235, 279, 319	–	–	–	2.48	–	–	–	–	–
Oleuropein derivative II	26.97	235, 280, 319	–	–	–	1.74	–	–	–	–	–
Oleuropein	27.27	238, 281, 318	–	–	–	**4.67**	–	–	–	–	–
Oleuropein derivative III	28.95	236, 276	–	–	–	2.15	–	–	–	–	–
**Furanocoumarins**											
Psoralen	32.66	251, 310, 322, 333	–	–	I	–	–	–	–	–	–
8-Methoxypsoralen	32.96	238, 293	–	–	I	–	–	–	–	–	–
5-Methoxypsoralen	35.68	234, 268, 310	–	–	I	–	–	–	–	–	–
Total phenolic acids (derivatives)			7.09^c^	7.46^b^	5.16^d^	0.72^f^	1.31^e^	0.79^f^	16.22^a^	0.73	**
Total flavonoids (derivatives)			14.35^d^	8.42^e^	37.65^b^	5.96–	49.21^a^	15.04^c^	4.83^g^	3.48	**
Total tannins			–	–	–	–	–	–	115.66	–	–
Total phenylethanoids			–	4.56^b^	0.19^c^	12.06^a^	–	–	–	4.12	*

^1^Concentrations of individual phenolics are expressed as milligram per gram of plant dry weight (mg g^−1^ DW), followed by different superscript lowercase letters reporting significant differences between different plant extracts at *significant at *p* < 0.01; **significant at *p* < 0.001, according to Tukey’ s multiple range test.

^2^Data are presented as mean values (n = 3) with the determination of the least significant difference (LSD) for a *p* value < 0.05.

^3^Symbol “–” represents the compounds that were not detected or were detected in trace.

^4^Values in bold represent the major compounds identified in each plant species.

^5^“I” represents the compounds that were only identified by literature.

As shown in Table 2 and Fig. S1, phenolic profile of diverse plants was significantly different. Leaf extracts of both sage and rosemary were characterized by a high proportion of rosmarinic acid (4.61 mg g^−1^ or 4.31 mg g^−1^, respectively). Rue presented the highest content of rutin (26.10 mg g^−1^), followed by epicatechin gallate (7.82 mg g^−1^). The major phenolic components in olive leaves were oleuropein and its derivatives. Flavanones, especially eriodictyol glycosides, following rutin were found as predominant in the leaf and stem extracts of peppermint. Parsley was described in high amount of apigenin-7-*O*-apiosylglucoside also called apiin (4.04 mg g^−1^) and epicatechin (3.72 mg g^−1^) in its leaf and stem extracts. The principal phenolic constituents in pomegranate leaves were hydrolyzable tannins, particularly ellagitannin I (56.06 mg g^−1^) and ellagitannin II (45.16 mg g^−1^), ranking the highest concentrations among all identified compounds.

On the other hand, results from Table 2 and Fig. S1 also showed that the most abundant phenolic classes in the tested samples were phenolic acids, flavonoids, tannins, and phenylethanoids. A considerable variation of phenolics was found, ranging, for instance, from 0.03 mg g^−1^ of 2,3-hydrocybenzoic acid to 56.06 mg g^−1^ of ellagitannin I. For each identified compound, significant differences were observed (*p* < 0.05), such as gallocatechin. The most widespread phenolic acids present in the studied samples included hydroxybenzoic acids (gallic acid and its derivative, vanillic acid), hydroxycinnamic acids (caffeic acid, chlorogenic acid, and neochlorogenic acid), and their ester derivatives (e.g. rosmarinic acid). Significantly high contents of chlorogenic acid (1.54 mg g^−1^) and neochlorogenic acid (1.96 mg g^−1^), and the presence of coumaric acid were perceptible in rue extracts. Ellagic acid and its derivatives were abundant in pomegranate leaves. Except in sage and rosemary, rosmarinic acid was also found in peppermint (0.23 mg g^−1^), but its concentration was lower than that in literature^43^. The special existence of rosmarinic acid, rosmanol, epirosmanol, carnosol, and carnosic acid in sage and rosemary was consistent with other authors^23,25,44,45^.

Besides flavanols including gallocatechin, catechin and epicatechin gallate, then various flavones (luteolin and apigenin) and flavonols (quercetin and diosmetin), mainly in the forms of their derivatives were widely distributed in the most of the studied species. Among them, the highest content of gallocatechin (2.10 mg g^−1^), catechin (3.61 mg g^−1^) and epicatechin gallate (7.82 mg g^−1^) was detected in parsley, rosemary, and rue, respectively. Epicatechin was only found in parsley with a good quantity (3.72 mg g^−1^). Furthermore, the main flavonoids from our data present in sage, rosemary, rue, peppermint, and parsley were apigenin glycosides, luteolin glycosides, quercetin glycosides, flavanone glycosides, and apigenin glycosides, respectively. In addition, peppermint also had comparative amounts of luteolin and quercetin glycosides. Likewise, pomegranate leaves possessed several apigenin and luteolin glycosides. Particularly, rutin presented the highest proportion (26.10 mg g^−1^) in rue, followed by peppermint (9.90 mg g^−1^), while the lowest (0.86 mg g^−1^) in olive leaf.

An important observation is that pomegranate leaf extracts held the greatest number of hydrolyzable tannins, especially ellagitannins. Nevertheless, no ellagitannins were detected by the HPLC method in the other six plants, while condensed tannins were present by the spectrophotometric approach. This was possibly caused by the lack of authentic standards involving different tannins, which need to be performed in the chromatographic analysis. In practice, certain studies have reported the tannins present in sage, rosemary, peppermint, rue, parsley, and olive leaves^12,18,19,26,46–48^, mainly in the form of condensed tannins.

In some cases, phenylethanoids, which are phenethyl alcohol-structured phenolic antioxidants, were abundantly found in olive leaves, including oleuropein and its derivatives, followed by tyrosol and verbascoside. These molecules may conduct to its high antioxidant properties^11,49^. Tyrosol existed in highest concentration in rosemary (4.56 mg g^−1^), but in small quantity in olive leaf (0.75 mg g^−1^) and rue (0.19 mg g^−1^).

Many studies have described the domination of rosmarinic acid in sage and rosemary, detected in varied amounts depending on phenophase, genotypes, extraction methods, and geographical conditions^23–25,36,44,45,50^. A concentration ranging from 0.27 to 2.49% of rosmarinic acid was determined in rosemary leaf extract, according to regions^44^. Khaleel et al. ^45^ reported 4.5 µg mL^−1^ of rosmarinic acid in aqueous extract of rosemary, whereas 17.3 µg mL^−1^ was measured in our methanolic extract of this plant. Exceptionally high content of rosmarinic acid was found in May extract (19.375 mg L^−1^) of sage leaves described by Generalić et al. ^36^, very close to our data (18.653 mg L^−1^). Roby et al. ^23^ declared that the predominant phenolic compounds in sage methanolic extract were ferulic acid (18%), rosmarinic acid (17%) and apigenin (14%) of the total extracted phenols, while in our results, rosmarinic acid and apigenin glycoside III were primary and accounted for 9% and 13% of the total phenolics of sage. Among more than one-hundred active ingredients of rue, rutin, as one of its major compounds, has been a topic of interest for researchers^9,20^. Asgharian et al. ^20^ detected a high level of rutin (40.15 mg g^−1^) by extraction with 70% ethanol, which was higher than that of our study. Melnyk et al. ^46^ identified rutin as the highest content of phenolics in the rue methanolic extract, consistent with the present work. Several studies^48,49^ have reported oleuropein and its derivatives as the dominant phenolics in the olive leaf, according with our results. As shown in data (Table 2 and Fig. S1), up to 20 phenolic compounds were identified in methanolic extract of olive leaf, more than those identified in other six plants, evidencing it as a rich source of bioactive compounds. However, the composition of olive leaf shows a remarkable variability due to location, climatic-seasonal factors, and cultivation practices, suggesting a trend to understand the factors that control the composition of olive leaves. This can be worthy for the harvesting and production of suitable extracts to be applied in human health. Kapp et al. ^43^ demonstrated eriocitrin, as a powerful bioactive compound, was the most abundant phenolics in peppermint, in accord with our records, composed of 38% of its aqueous extract, or reaching from 19.9 to 68.1% in 26 peppermint tea samples, respectively. However, the same authors^43^ reported that rosmarinic acid accounted for a highest proportion (54.2%) of phenolics in one peppermint tea sample which was originated from Estonia. Additionally, other authors^22,47,51^ also pointed out different dominant phenolics in peppermint, such as epicatechin, naringenin, caffeic acid, chlorogenic acid, 4-hydroxybenzoic acid, which can be attributed to diverse varieties, growing environment, and extraction conditions. The main finding of the present work performed on parsley corresponded to several studies^15,52^ that apiin extractability was maximum when the solvent was ethanol, methanol or acetone. Yet Hozayen et al. ^53^ and Aissani et al. ^21^ conducted rosmarinic acid and quinic acid as the most abounded constituent in aqueous and methanol extracts of parsley, respectively. Fourteen phenolic constituents (Figure S1) of pomegranate leaf extracts were preliminarily identified and quantified by reference to chromatographic parameters and the literature. These results are agreeable to other researchers^33,54–56^, highlighting that ellagic acid and its derivatives, ellagitannins (punicalin, granatin A and B, etc.), flavone (apigenin, luteolin) and its glycosides, and flavonol (kaempferol) and its glycosides, are the principal phenolics in pomegranate leaves. In addition, many ellagitannins (such as punicalagins, punicafolin, castalagin, corilagin, strictinin, tercatain, brevifolin), and their galloyl and/or hexahydroxydiphenoyl (HHDP) substitutions, have been isolated from the leaf^57^. Other flavonoid derivatives like kaempferol, gossypin, quercetin, and rutin were also detected as major constituents in hydro-methanolic or hydro-ethanolic leaf extracts of pomegranate leaves^33,57^. However, the detailed structures of tannins and flavonoids of pomegranate leaf will require further identification by mass spectrometry and nuclear magnetic resonance spectroscopy.

### Correlation analysis

**Figure 1:**
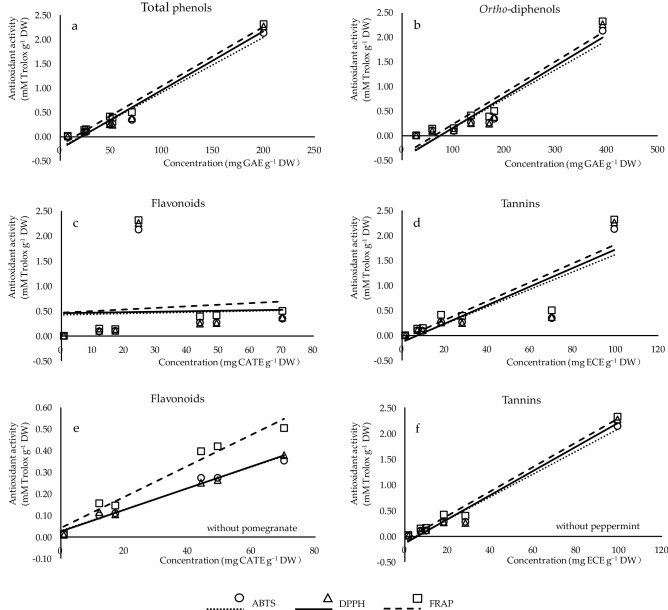
Correlation analysis between the contents of phenolic classes (x-axis) and antioxidant capacities (y-axis) measured by ABTS (circles), DPPH (triangles), and FRAP (squares). (**a**–**d**) The correlation of total phenols (*r*_ABTS_, _DPPH_, _FRAP_ = 0.985***, 0.984***, 0.993***), *ortho*-diphenols (*r*_ABTS_, _DPPH_, _FRAP_ = 0.859*, 0.861*, 0.878**), flavonoids (*r*_ABTS_, _DPPH_, _FRAP_ = 0.038, 0.031, 0.098), and tannins (*r*_ABTS_, _DPPH_, _FRAP_ = 0.859*, 0.861*, 0.878**) of the studied plants with their antioxidant activity, respectively. (**e**) The correlation of flavonoids (*r*_ABTS_, _DPPH_, _FRAP_ = 0.989***, 0.992***, 0.983***) of studied plants excluding pomegranate with their antioxidant activity. (**f**) The correlation of tannins of studied plants excluding peppermint (*r*_ABTS_, _DPPH_, _FRAP_ = 0.989***, 0.987***, 0.993***) with their antioxidant activity.

In order to better understand the relationship between the antioxidant activity (by ABTS, DPPH, FRAP assays) and the phenolic composition (total phenols, *ortho*-diphenols, flavonoids, tannins) of the studied plants, correlation coefficients (*r*) were determined (Fig. 1). Strong relationships were characterized between antioxidant capacities with total phenols and *ortho*-diphenols (Fig. 1a,b), indicating that phenolic compounds contribute to the inhibition of oxidative processes. The content of tannins was well correlated with antioxidant potential (Fig. 1d). No correlation of antioxidant activities was found with flavonoid content (Fig. 1c). However, a better relationship of flavonoids (Fig. 1e) or tannins (Fig. 1f) can be obtained with the antioxidant activity if excluding pomegranate or peppermint from the data, respectively. The above analysis demonstrated that the antioxidant potential from different plants was dependent on both the concentrations and the structures of phenolic compounds, in line with Cai et al. ^30^. Compared to radical scavenging assays (ABTS and DPPH), the stronger correlation between reducing power and phenolic contents confirmed that FRAP was more closely related to total phenols, *ortho*-diphenols and tannins, which was also mentioned by Li et al. ^1^.

There is a highly correlation between the phenolic composition and antioxidant properties of plants. High anti-radical activity of rosemary leaf in summer was strongly related to high amounts of total phenols, total flavonoids, condensed tannins, and carnosic acid^18^. It is suggested that intraperitoneal of hydroalcoholic extract of rue increased serum and brain antioxidant capacity, due to their potent antioxidant activities of total phenolic and flavonoids content, especially rutin, caffeic acid, and apigenin^20^. Parsley methanolic extract inhibited human glioblastima cancer and oxidative stress owing to its antioxidant properties primarily related to phenolic content^21^. Peppermint extracted by various alcoholic solvents are found to have different levels of antioxidant potential, attributed to the presence of vast flavonoids, anthocyanins, and total phenols^22^. The strong reducing power, free radical scavenging capacity, and the inhibition of hydro-peroxide radicals activity of sage leaves can be linked to the high quantity of phenolic acids, especially rosmarinic acid, and certain flavonoids like catechins and flavanols^36^. Makowska-Wąs et al. ^49^ revealed considerable antioxidant and cytotoxic properties of olive leaf against several human cancers, largely concerned in the existence of phenolic acids, flavonoids, oleuropein, fatty acids, and volatile oils. The high concentration of phenolic components in pomegranate leaf extracts such as tannins, flavonoids, phyto-steroids, terpenoids, and saponins can be responsible for its high antioxidant activity in vitro and in vivo^27–29,32,58^.

To date, amount of studies have reported the close relationship not only between the phenolic contents but also between the phenolic structures and the antioxidant capacities^28,30,59^. The level of antioxidant potential of plants mainly depends on the presence and hydroxyl groups of (poly)phenolic compounds. Specifically, the antioxidant ability of phenolic acids is firstly related to the number and position of phenolic hydroxyls, and secondly to the methoxy and carboxylic acid groups^59^. Rosmarinic acid which was mainly detected in sage, rosemary, and peppermint in our work, is an ester of caffeic acid and 3,4-dihydroxyphenyl lactic acid, comprising two catechol moieties, thus having two pairs of *ortho* hydroxyl groups grafted on two phenolic rings^18^. Gallic and chlorogenic acid are well-known antioxidant agents, due to three and two active hydroxyl groups on the aromatic ring, respectively^59^. Moreover, the catechol structure in the B-ring, the 2,3-double bond conjugated to a 4-oxo functionality, and the available of both 3- and 5-hydroxyl groups of flavonoids are essential for assessing their antioxidant properties^28^. Rutin is a rutinoside of quercetin with one of the four hydroxyl groups at position C-3 substituted with glucose and rhamnose sugar groups^20^. Apiin or eriocitrin is a apigenin or eriodictyol glycoside, on which the different glycoside moiety is located at position C-7 via a glycosidic linkage along with two or three residual hydroxyl groups on the phenolic rings^15,43^. Furthermore, phenylethanoids are characterized by a phenethyl alcohol (C6–C2) moiety attached to a β-glucopyranose/β-allopyranose via a glycosidic bond. Studies indicated the *ortho*-dihydroxyphenyl groups were the most significant, and the steric hindrance, the number and the position of phenolic hydroxyls were also thought to play an important role^60^. Oleuropein with two hydroxyl groups is an ester of elenolic acid and hydroxytyrosol, and has a oleosidic skeleton that is common to the secoiridoid glucosides of *Oleaceae*^49^. The strong correlation of antioxidant property with well-identified phenolic acids, flavonoids, and oleuropein present in sage, rosemary, peppermint, rue, parsley, and olive leaves has been individually demonstrated to explain their diverse biological functions^6–9,11,13^. In addition, ellagic acid and tannins, defined as polyphenols, are complex chemical substances, possessing plentiful hydroxyl groups, especially *ortho*-dihydroxyl or galloyl groups^61^. Bigger tannin molecules appear more galloyl and *ortho*-dihydroxyl groups, consequently, their activities are stronger^61^. Ellagitannins, ellagic acid, and their metabolites have been reported to exhibit numerous beneficial effects on human health including antioxidant, anti-inflammatory, anti-cancer, prebiotic, and cardio-protective properties^61^. Thus they deserve to be part of a healthy diet as functional foods.

The researches on the structure–activity relationship between phenolics and their antioxidant activities have focused on phenolic acids and flavonoids, as well as oleuropein and its derivatives owing to their partially acknowledged health-promoting effects^2,30^. However, the benefits of medicinal and food plants may arise from the action of some less well-studied antioxidant molecules or from a synergy of certain antioxidants^30^. Cai et al. ^30^ found some anticancer-related medicinal plants contained higher quantities and more sorts of tannins, quinones, phenolic terpenoids and special phenolic glycosides than that of phenolic acids and flavonoids. Regarding pomegranate leaves, some authors detected kaempferol^54^ or kaempferol 3-*O*-glycoside^33^ as the main compound in ethanolic extracts, while others found as ellagic acid^55^. The principal ellagitannins of pomegranate leaves also differed from one another, considered as granatin B^56^, or castalagin derivative^33^, or undefined galloy-HHDP derivatives^55^. This difference may be induced by varieties, phenology, and growing conditions. In our study, the potent antioxidant capacity of pomegranate leaves was highly correlated with the content of tannins, which can be considered as the key antioxidant contributors of this plant material. However, the chemical structures of the tentatively identified ellagitannins were not determined, and studies on these constituents are also incomplete. Therefore, it is important to note although this is a preliminary study to provide a baseline of data for future investigations, a major limitation is that identified phyto-constituents were neither isolated, nor separately analyzed for their bioactivities. Moreover, the association between these compounds and antioxidant effect of pomegranate leaf is yet to be well understood. In this regard, it is necessary to further characterize the structure of these less-exploited phenolics (tannins) and their associated biological properties within pomegranate leaf. Hence, the results presented in our study confirm pomegranate leaf as a promising natural alternative in the development of antioxidant products, thereby assisting in the prevention and treatment of some diseases.

## Conclusions

The level of different phenolic classes, antioxidant capacities and the phenolic profiles of seven medicinal and food plants were evaluated and correlated, including the leaves of sage, rosemary, olive, and pomegranate, as well as the leaves and young stems of rue, peppermint, and parsley. This study compared and demonstrated these plant extracts as valuable sources of bioactive compounds, likely for preparing novel functional products in various industries. High correlations of phenolic composition with antioxidant potential were investigated in our analysis. Different kinds of phenolic acids and flavonoids along with their derivatives were found widespread in the studied plant materials. Phenylethanoids especially oleuropein and its derivatives were characterized as the most abundant constituents of olive leaf extracts, probably contributing to its beneficial biological properties. While tannins particularly ellagitannins were supposed to be the main contributor to the features of pomegranate leaf. Interestingly, our results highlighted that the hydro-methanolic extracts of *Punica granatum* L. (pomegranate) leaves displayed the greatest levels of free radical scavenging capacity and ferric reducing antioxidant power, as well as the highest contents of total phenols, *ortho*-diphenols and tannins; a relatively high content of flavonoids was also found. Studies have increasingly evidenced the close association of tannins and less-studied compounds with antioxidant activity in medicinal and food plants^12,18,19,26,48^. Thus it is expected that richer phenolic types, namely tannins and phenolic glycosides, and their higher concentrations, are maintained in pomegranate leaves, making it possible to explore active ingredients and bioavailable products in the food-pharm, nutraceutical or cosmeceutical industries.

Moreover, only a limited number of researches have pointed out the comparison of biological activities and phenolic components of the tested plant organs, which belong to tree plants or shrub plants with large or small leaves. Many authors have stated the importance of vegetables, fruits, medicinal and aromatic plants in the current dietary patterns^2–5,29,30,50^. However, it doesn’t mean the agricultural and industrial waste like the tree leaves are useless for application. Extracts of olive leaves have attracted more attention recently, being reviewed as promising cheap, renewable and plenty source of bio-phenols for by-products. Some articles proved pomegranate leaf as a safe substrate due to its lower or inexistent toxicity^17,35^. In addition, ellagitannins as effective ingredients in teas are considered to be more abundant in the large-leaf tree than those from the small-leaf tree^61,62^. Therefore, as per olive leaf, research into finding new uses for by-products of pomegranate leaf may be proved as a strong argument for not only promoting human health but also improving bio-valorization and environment. However, samples of pomegranate leaves were not collected from different varieties or different seasons. Hence, studies on these issues would be of much interest in the future, in order to select the most promising matrix of the wasted bio-phenol materials.

## Materials and methods

### Chemicals and standards

Compounds: 2,2′-azino-bis (3-ethylbenzothiazoline-6-sulfonic acid) diammonium salt (ABTS^·+^), (±)-6-hydroxy-2,5,7,8-tetramethylchromone-2-carboxylic acid (Trolox), 2,2-diphenyl-1-picrylhidrazyl radical (DPPH^·^), 2,4,6-tris(2-pyridyl)-s-triazine (TPTZ), sodium carbonate, sodium molybdate, potassium persulfate, and hydrochloric acid, all extra pure (> 99%) were obtained from Sigma-Aldrich (Sigma-Aldrich, St. Louis, MO, USA). Reagents: ferric chloride, methanol, aluminum chloride, sodium nitrite, all extra pure (> 99%), and methyl cellulose (1500 centipoises viscosity at 2%) were acquired from Merck (Merck, Darmstadt, Germany). Sodium hydroxide, ammonium sulfate, Folin-Ciocalteu’s reagent and acetic acid, all extra pure (> 99%) were purchased from Panreac (Panreac Química S.L.U., Barcelona, Spain). Authentic standards of phenolic compounds used in the chromatographic analysis, including that protocatechuic acid (> 97%), *p*-hydroxybenzoic acid (> 99%), benzoic acid (> 99.5%) were obtained from Fluka (Fluka Chemika, Neu-Ulm, Switzerland), and caffeic acid (> 98%) was from Panreac (Panreac Química S.L.U., Barcelona, Spain). Standards: neochlorogenic acid (> 95%), chlorogenic acid (> 99%), vanillic acid (> 97%), syringic acid (≥ 99%), myricitin-3-*O*-glucoside (≥ 99%), *p*-coumaric acid (> 99%), rutin (quercetin-3-rutinoside) (≥ 94%), ellagic acid (≥ 95%), ferulic acid (> 99%), apigenin-7-*O*-glucoside (≥ 95%), rosmarinic acid (≥ 98%), luteolin (≥ 98%), quercetin (> 95%), *trans*-cinnamic acid (> 95%), and kaempferol (> 90%) were purchased from Chem-Lab (Chem-Lab N.V., Zedelgem, Belgium). Gallic acid (> 97.5%), tyrosol (> 98%), caftaric acid (≥ 97%), catechin (≥ 98%), gentisic acid (≥ 98%), epicatechin (≥ 98%), 4-hydrocinnamic acid (> 95%), luteolin-7-*O*-glucoside (≥ 98%), isorhamnetina-3-*O*-glucoside (> 95%), oleuropein (> 98%), resveratrol (≥ 99%), and *trans*-stilben (> 96%) were acquired from Sigma-Aldrich (Sigma-Aldrich, St. Louis, MO, USA). Chromatography solvents were of RP-HPLC-DAD grade according to the analysis performed. Ultrapure water was obtained using a Water Purification System (Arioso Power, Human Corporation, Seoul, Korea).

### Plant materials

From about one-hundred common medicinal and food plants reported in literature references, we have selected seven medicinal and food plants (Table S1) in this study according to following criteria: (1) higher phenolic content and antioxidant capacity, (2) lower or inexistent toxicity. Plant species were botanically authenticated by Prof. António Crespí (Department of Biology and Environment, University of Trás-os-Montes e Alto Douro, UTAD, Portugal) and Dr. João Rocha (Chemistry Centre-Vila Real, UTAD, Portugal). Samples of each species were hand-picked randomly from a pool of individual specimens (n > 10) that are naturally growing in the Botanical Garden of UTAD (Vila Real, Portugal), which belongs to the international network of botanical gardens. Sage, rosemary, rue, peppermint, and parsley are present in the Aromatic and Medicinal Plants collection; olive is present in the Mediterranean Calcareous collection; pomegranate is present in the Garden Fruits collection (more detailed information of each plant species can be checked at http://jb.utad.pt/). Thus, a mixture sample for each species was obtained and used for the subsequent analysis. The collected samples were immediately dried at 40 ℃ (Drying Cabinet, LEEC, Nottingham, UK) for 72 h, before being ground into a fine powder with a blender (MB 800, KINEMATICA AG, Malters, Switzerland), and hermetically stored in the dark, at room temperature (RT) until analysis. Experimental research and field studies on plants (either cultivated or wild), including the collection of plant material have complied with relevant institutional, national, and international guidelines and legislation.

### Preparation of plant phenolic extracts

The sample powder of each species was weighed and extracted in triplicate with 40 mg of dry weight (DW). The extraction was performed by agitating (30 min, 200 rpm, RT) the mixture of the powder and 1.5 mL of a hydro-methanolic solution (methanol:H_2_O, 70:30, v/v) in an orbital shaker (GFL 3005, GEMINI, Apeldoorn, Netherlands). Afterwards, the suspensions were centrifuged (10,000 rpm, 4 ℃) for 15 min (Sigma 2-16KL Refrigerated Centrifuges, Sigma Laborzentrifugen, Berlin, Germany). The supernatants were collected in a 5 mL volumetric flask, and the solid residues were then extracted twice via the same procedure. All the three supernatants from successive extractions were kept together and the final volume came to 5 mL with the above-mentioned extraction solvent.

### Content of different phenolic classes

The content of total phenols, *ortho*-diphenols, and flavonoids was determined by colorimetric and spectrophotometric approaches according to the literature^63^. The content of tannins was evaluated by the methyl cellulose (MC) methodology previously reported by Dambergs et al. ^64^.

For the determination of total phenol content, 20 μL of diluted sample, 100 μL of diluted Folin-Ciocalteu reagent (90%, v/v), and 80 μL aqueous sodium carbonate (7.5%, w/v) were mixed in sequence. The mixture was incubated for 30 min at 42 ℃ in the dark and measured at 750 nm, using gallic acid as standard. Results were expressed in milligrams of gallic acid equivalents per gram of plant dry weight (mg GAE g^−1^ DW).

For the assessment of *ortho*-diphenols content, 40 μL of sodium molybdate solution (5%, w/v) prepared with hydro-methanol (50%, v/v) was added to 160 μL of diluted extract. The mixture was stood for 15 min at RT, protected from light, before the absorbance at 375 nm was read. The content was quantified using gallic acid as standard. Results were defined in mg GAE g^−1^ DW.

For the quantification of total flavonoids content, 24 μL of diluted extract and 28 μL of sodium nitrite (5%, w/v) were mixed. After 5 min at RT, 28 μL of a 10% (w/v) aluminum chloride solution was added in the mixture and reacted for 6 min. Afterwards, 120 μL of sodium hydroxide (1 M) was added and the final mixture was read at 520 nm after agitation for 30 s in a microplate reader. The results were expressed in milligrams of catechin equivalents per gram of plant dry weight (mg CATE g^−1^ DW).

The above-mentioned assays were undertaken with a microplate reader (Multiskan FC Microplate Photometer, Thermo Fisher Scientific, Vantaa, Finland) in 96-well microplates (PrimeSurface MS-9096MZ, Frilabo, Maia, Portugal) with a final volume of 200 µL.

The content of tannins was evaluated both in treatment and control groups simultaneously, by adding 600 μL of methyl cellulose (MC) solution (treatment) or water (control) to 200 μL of sample in a 2 mL Eppendorf. The mixture was stirred manually for 2–3 min at RT. Four hundred μL of saturated ammonium sulfate and 800 μL of water were added successively both in the treatment and control groups until 2 mL of total volume was reached. The final mixture was vortexed and kept for 10 min. After centrifugation (10,000 rpm, 16 ℃, 5 min), the absorbance was read at 280 nm, by using a conventional spectrophotometer (Helios Gamma UV Spectrophotometer, Thermo Electron Corporation, Warwickshire, UK). The absorbance of tannins was obtained by subtracting the treatment absorbance from the value registered from the control, using epicatechin as standard. The results were described in milligrams of epicatechin equivalents per gram of plant dry weight (mg ECE g^−1^ DW).

### Evaluation of in vitro antioxidant activity

The antioxidant activity of sample extracts was determined by ABTS, DPPH and FRAP (ferric reducing antioxidant power) spectrophotometric methods, reported by Mena et al. ^65^, with some modifications.

The ABTS^+^ radicals were produced by mixing 5 mL of ABTS stock solution (7.0 mM) with 88 μL of potassium persulfate (148 mM), and diluted to a working solution with sodium acetate buffer (20 mM, pH 4.5), showing an absorbance of 0.70 ± 0.02 at 734 nm. Subsequently, 188 μL of ABTS working solution and 12 μL of sample dilutions (water used as blank) were mixed and reacted for 30 min at RT, and then the absorbance was read at 734 nm.

The DPPH radicals (8.87 mM) were formed with methanol (99.9%) and diluted in a working solution with hydro-methanol (70%, v/v), achieving an absorbance of 1000 at 520 nm. A mixture of 190 μL of DPPH working solution and 10 μL of sample dilutions (70% hydro-methanol used as blank) was incubated for 15 min at RT, reading the absorbance at 520 nm.

The FRAP working solution was prepared by mixing 10-volume acetate buffer (300 mM, pH 3.6), 1-volume TPTZ (10 mM dissolved in hydrochloric acid), and 1-volume ferric chloride (20 mM in water). The mixture was maintained at 37 ℃ for 10 min before use. The reaction of FRAP working solution (180 μL) and sample dilutions (20 μL) was kept at 37 ℃ for 30 min and the absorbance read at 593 nm.

The three antioxidant assays were adapted to microscale using 96-well microplates (PrimeSurface MS-9096MZ, Frilabo, Maia, Portugal) and microplate readers (Multiskan GO Microplate Photometer, Thermo Fisher Scientific, Vantaa, Finland), using Trolox as standard. All the results were expressed in millimoles of Trolox per gram of plant dry weight (mM Trolox g^−1^ DW).

### Chromatographic analysis of phenolic compounds

Reverse phase-high performance liquid chromatography-diode array detector (RP-HPLC-DAD) system (Thermo Finnigan, San Diego, CA, USA) was carried out to determine the (poly)phenolic profile of each plant extract, as previously described^63^. The analysis equipment is composed of three parts, including LC pump (Surveyor), autosampler (Surveyor), and PDA detector (Surveyor). Sample extracts, in triplicate, and 31 pure standard compounds (all in HPLC grade), including 17 phenolic acids, 10 flavonoids, 2 phenylethanoids and 2 stilbenoids, were prepared and filtered through 0.45 μm PVDF filters (Millex-HV Syringe Filter Unit, Merck Millipore, Bedford, MA, USA) and injected into a C18 column (250 × 4.6 mm, 5 μm particle size; ACE, Aberdeen, Scotland), using a mobile phase composed of water/formic acid (99.9:0.1, v/v) (solvent A) and acetonitrile/formic acid (99.9:0.1, v/v) (solvent B). The linear gradient program (t in min and %B) was: t = 0–0%; t = 5–0%; t = 20–20%; t = 35–50%; t = 40–100%; t = 45–0%; and t = 65–0%. The injection volume was 20 μL and the flow rate was kept at 1.0 mL min^−1^. UV/Vis detection was recorded from 200 to 600 nm range. Peaks were monitored at 280 and 330 nm, and identified by congruent retention time compared with standards. Data acquisition, peak integration and analysis were performed using Chromeleon software (Version 7.1; Thermo Scientific, Dionex, USA). The three extracts of each medicinal plant were chromatographed and results were expressed in milligram per liter of sample extracts (mg L^−1^).

### Data and statistical analysis

All the measurements of phenolic phytochemicals and antioxidant activity of the plant extracts were conducted in triplicate. The results of phenolic content and antioxidant activity are presented as mean ± standard deviation (SD). Concentrations of individual identified phenolic compounds are presented as mean (n = 3) with the determination of the Least Significant Difference (LSD) for a *p* value < 0.05. The obtained data were subjected to analysis of variance (ANOVA) and a multiple range test (Tukey’s test) with IBM SPSS statistics 21.0 software (SPSS Inc., Chicago, USA). Pearson (*r*) analysis was carried out to establish correlations between phenolic chemical classes and antioxidant activity.

## Supplementary Information

Supplementary Information 1.

Supplementary Information 2.

## References

[1] Li Q (2017). Cholinesterase, *β*-amyloid aggregation inhibitory and antioxidant capacities of Chinese medicinal plants. Ind. Crops Prod..

[2] Nollet LM, Gutierrez-Uribe JA (2018). Phenolic Compounds in Food: Characterization and Analysis.

[3] Uritu CM (2018). Medicinal plants of the family Lamiaceae in pain therapy: A review. Pain Res. Manag..

[4] Etkin NL (2019). Plants and Indigenous Medicine and Diet: Biobehavioral Approaches.

[5] Watson RR, Preedy VR (2016). Fruits, Vegetables, and Herbs: Bioactive Foods in Health Promotion.

[6] Alavi MS, Fanoudi S, Ghasemzadeh Rahbardar M, Mehri S, Hosseinzadeh H (2020). An updated review of protective effects of rosemary and its active constituents against natural and chemical toxicities. Phytother. Res..

[7] Ghorbani A, Esmaeilizadeh M (2017). Pharmacological properties of *Salvia officinalis* and its components. J. Tradit. Complement. Med..

[8] Mahendran G, Rahman L-U (2020). Ethnomedicinal, phytochemical and pharmacological updates on Peppermint (*Mentha × piperita* L.)—a review. Phytother. Res..

[9] Shamal Badhusha PA (2020). Traditional uses, phytochemistry and ethanopharmacology of *Ruta graveolens* Linn: A review. Int. J. Pharm. Drug Anal..

[10] Colucci-D’Amato L, Cimaglia G (2020). *Ruta graveolens* as a potential source of neuroactive compounds to promote and restore neural functions. J. Tradit. Complement. Med..

[11] Şahin S, Bilgin M (2018). Olive tree (*Olea europaea* L.) leaf as a waste by-product of table olive and olive oil industry: A review. J. Sci. Food Agric..

[12] Salama ZA (2020). In-vitro antioxidant, antimicrobial and anticancer activities of banana leaves (*Musa acuminata*) and olive leaves (*Olea europaea* L.) as by-products. Res. J. Pharm. Technol..

[13] Farzaei MH, Abbasabadi Z, Ardekani MRS, Rahimi R, Farzaei F (2013). Parsley: A review of ethnopharmacology, phytochemistry and biological activities. J. Tradit. Chin. Med..

[14] Cefali LC (2019). Evaluation of in vitro solar protection factor (SPF), antioxidant activity, and cell viability of mixed vegetable extracts from *Dirmophandra mollis* Benth, *Ginkgo biloba* L., *Ruta graveolens* L., and *Vitis vinífera* L.. Plants.

[15] Mara de Menezes Epifanio N (2020). Chemical characterization and in vivo antioxidant activity of parsley (*Petroselinum crispum*) aqueous extract. Food Funct..

[16] Vučić V, Grabež M, Trchounian A, Arsić A (2019). Composition and potential health benefits of pomegranate: A review. Curr. Pharm. Des..

[17] Viswanatha GL, Venkataranganna MV, Prasad NBL, Ashok G (2016). Evaluation of anti-epileptic activity of leaf extracts of *Punica granatum* on experimental models of epilepsy in mice. J. Intercult. Ethnopharmacol..

[18] Yeddes W, Chalghoum A, Aidi-Wannes W, Ksouri R, Saidani Tounsi M (2019). Effect of bioclimatic area and season on phenolics and antioxidant activities of rosemary (*Rosmarinus officinalis* L.) leaves. J. Essential Oil Res..

[19] Christova-Bagdassarian VL, Bagdassarian KS, Atanassova MS, Ahmad MA (2014). Comparative analysis of total phenolic and total flavonoid contents, rutin, tannins and antioxidant capacity in Apiaceae and Lamiaceae families. Indian Hortic. J..

[20] Asgharian S (2020). *Ruta graveolens* and rutin, as its major compound: Investigating their effect on spatial memory and passive avoidance memory in rats. Pharm. Biol..

[21] Aissani N, Albouchi F, Sebai H (2020). Anticancer effect in human glioblastoma and antioxidant activity of *Petroselinum crispum* L. methanol extract. Nutr. Cancer.

[22] Farnad N, Heidari R, Aslanipour B (2014). Phenolic composition and comparison of antioxidant activity of alcoholic extracts of Peppermint (*Mentha piperita*). J. Food Meas. Charact..

[23] Roby MHH, Sarhan MA, Selim KA-H, Khalel KI (2013). Evaluation of antioxidant activity, total phenols and phenolic compounds in thyme (*Thymus vulgaris* L.), sage (*Salvia officinalis* L.), and marjoram (*Origanum majorana* L.) extracts. Ind. Crops Prod..

[24] Dent M, Dragović-Uzelac V, Penić M, Bosiljkov T, Levaj B (2013). The effect of extraction solvents, temperature and time on the composition and mass fraction of polyphenols in Dalmatian wild sage (*Salvia officinalis* L.) extracts. Food Technol. Biotech..

[25] Mulinacci N (2011). Storage method, drying processes and extraction procedures strongly affect the phenolic fraction of rosemary leaves: An HPLC/DAD/MS study. Talanta.

[26] Ramkissoon JS, Mahomoodally MF, Ahmed N, Subratty AH (2013). Antioxidant and anti-glycation activities correlates with phenolic composition of tropical medicinal herbs. Asian Pac. J. Trop. Med..

[27] Mestry SN, Dhodi JB, Kumbhar SB, Juvekar AR (2017). Attenuation of diabetic nephropathy in streptozotocin-induced diabetic rats by *Punica granatum* Linn. leaves extract. J. Tradit. Complement. Med..

[28] Uysal S, Zengin G, Aktumsek A, Karatas S (2016). Chemical and biological approaches on nine fruit tree leaves collected from the Mediterranean region of Turkey. J. Funct. Foods.

[29] Kaewnarin K, Niamsup H, Shank L, Rakariyatham N (2014). Antioxidant and antiglycation activities of some edible and medicinal plants. Chiang Mai J. Sci..

[30] Cai Y, Luo Q, Sun M, Corke H (2004). Antioxidant activity and phenolic compounds of 112 traditional Chinese medicinal plants associated with anticancer. Life Sci..

[31] Elfalleh W (2012). Total phenolic contents and antioxidant activities of pomegranate peel, seed, leaf and flower. J. Med. Plants Res..

[32] Fellah B (2020). Untargeted metabolomics reveals changes in phenolic profile following in vitro large intestine fermentation of non-edible parts of *Punica granatum* L.. Food Res. Int..

[33] Pinheiro AJMCR (2018). *Punica granatum* L. leaf extract attenuates lung inflammation in mice with acute lung injury. J. Immunol. Res..

[34] Ulewicz-Magulska B, Wesolowski M (2019). Total phenolic contents and antioxidant potential of herbs used for medical and culinary purposes. Plant Food Hum. Nutr..

[35] Ankita P, Deepti B, Nilam M (2015). Flavonoid rich fraction of *Punica granatum* improves early diabetic nephropathy by ameliorating proteinuria and disturbed glucose homeostasis in experimental animals. Pharm. Biol..

[36] Generalić I (2011). Influence of the phenophase on the phenolic profile and antioxidant properties of Dalmatian sage. Food Chem..

[37] Salwe KJ, Sachdev D (2014). Evaluation of antinociceptive and anti-inflammatory effect of the hydroalcoholic extracts of leaves and fruit peel of *P. granatum* in experimental animals. Asian J. Pharm. Clin. Res..

[38] AlFadel F, Al Laham S, Alkhatib R (2014). The anti-bacterial activity of various parts of *Punica granatum* on antibiotics resistance *Escherichia coli*. Seeds.

[39] Gheith I, El-Mahmoudy A (2017). Potent anti-oxidant and anti-inflammatory potentials of *Punica granatum* leaf and flower hydromethanolic extracts in vitro. Biosci. J..

[40] Pararin S, Rouhi L, Ghasemi Pirbalouti A (2016). The beneficial effect of hydro-alcoholic extract of *Punica granatum* L. leaves and flower on ethylene glycol-induced kidney calculi in RATS. J. Herb. Drugs (Int. J. Med. Herbs).

[41] Kiraz Y, Neergheen-Bhujun VS, Rummun N, Baran Y (2016). Apoptotic effects of non-edible parts of *Punica granatum* on human multiple myeloma cells. Tumour Biol. J. Int. Soc. Oncodev. Biol. Med..

[42] Rummun N, Somanah J, Ramsaha S, Bahorun T, Neergheen-Bhujun VS (2013). Bioactivity of nonedible parts of *Punica granatum* L.: A potential source of functional ingredients. Int. J. Food Sci..

[43] Kapp K (2013). Commercial peppermint (*Mentha × piperita* L) teas: Antichlamydial effect and polyphenolic composition. Food Res. Int..

[44] Meziane-Assami D, Tomao V, Ruiz K, Meklati BY, Chemat F (2013). Geographical differentiation of rosemary based on GC/MS and fast HPLC analyses. Food Anal. Method.

[45] Khaleel IR, Kadhim MI, Subhi JH (2018). Effect of some biotic and abiotic elicitors on phenolic acids and diterpenes production from rosemary (*Rosmarinus officinalis* L.) leaf and callus analyzed by high performance liquid chromatography (Hplc). Al-Nahrain J. Sci..

[46] Melnyk M, Vodoslavskyi V, Obodianskyi M (2018). Research of phenolic compounds of ruta graveolens l and stellaria media (l.) vill. Asian J. Pharm. Clin. Res..

[47] Figueroa-Pérez MG (2018). Diabetic nephropathy is ameliorated with peppermint (*Mentha piperita*) infusions prepared from salicylic acid-elicited plants. J. Funct. Foods.

[48] Dekanski D (2009). Phytochemical analysis and gastroprotective activity of an olive leaf extract. J. Serb. Chem. Soc..

[49] Makowska-Wąs J (2017). Identification of predominant phytochemical compounds and cytotoxic activity of wild olive leaves (*Olea europaea* L. ssp. sylvestris) harvested in South Portugal. Chem. Biodiv..

[50] Skotti E (2016). Biological activity of selected Greek medicinal and aromatic plants extracts on Alternaria alternata. Emirates J. Food Agric..

[51] Arruda MO (2017). The hydroalcoholic extract obtained from *Mentha piperita* L. leaves attenuates oxidative stress and improves survival in lipopolysaccharide-treated macrophages. J. Immunol. Res..

[52] Luthria DL, Mukhopadhyay S, Kwansa AL (2006). A systematic approach for extraction of phenolic compounds using parsley (*Petroselinum crispum*) flakes as a model substrate. J. Sci. Food Agric..

[53] Hozayen WG, El-Desouky MA, Soliman HA, Ahmed RR, Khaliefa AK (2016). Antiosteoporotic effect of *Petroselinum crispum*, *Ocimum basilicum* and *Cichorium intybus* L. in glucocorticoid-induced osteoporosis in rats. Bmc. Complement. Altern. Med..

[54] Marques LC (2016). Anti-inflammatory effects of a pomegranate leaf extract in LPS-induced peritonitis. Planta Med..

[55] Swilam N, Nematallah KA (2020). Polyphenols profile of pomegranate leaves and their role in green synthesis of silver nanoparticles. Sci. Rep..

[56] Akkawi M, Abu-Lafi S, Abu-Remeleh Q (2019). Phytochemical screening of Pomegranate juice, peels, leaves and membranes water extracts and their effect on β-hematin formation, a comparative study. Pharm. Pharmacol. Int. J..

[57] Pinheiro AC (2019). Galloyl-hexahydroxydiphenoyl (HHDP)-glucose isolated from *Punica granatum* L. leaves protects against lipopolysaccharide (LPS)-induced acute lung injury in BALB/c mice. Front. Immunol..

[58] Lakshminarayanashastry Viswanatha G, Venkatanarasappa Venkataranganna M, Lingeswara Prasad NB (2019). Methanolic leaf extract of *Punica granatum* attenuates ischemia-reperfusion brain injury in Wistar rats: Potential antioxidant and anti-inflammatory mechanisms. Iran. J. Basic Med. Sci..

[59] Chen J (2020). Structure-antioxidant activity relationship of methoxy, phenolic hydroxyl, and carboxylic acid groups of phenolic acids. Sci. Rep..

[60] Xue Z, Yang B (2016). Phenylethanoid glycosides: Research advances in their phytochemistry, pharmacological activity and pharmacokinetics. Molecules (Basel, Switzerland).

[61] Fraga-Corral M (2020). Technological application of tannin-based extracts. Molecules.

[62] Yang X, Tomás-Barberán FA (2019). Tea is a significant dietary source of ellagitannins and ellagic acid. J. Agric. Food Chem..

[63] Gouvinhas I (2018). Monitoring the antioxidant and antimicrobial power of grape (*Vitis vinifera* L.) stems phenolics over long-term storage. Ind. Crops Prod..

[64] Dambergs RG, Mercurio MD, Kassara S, Cozzolino D, Smith PA (2012). Rapid measurement of methyl cellulose precipitable tannins using ultraviolet spectroscopy with chemometrics: Application to red wine and inter-laboratory calibration transfer. Appl. Spectrosc..

[65] Mena P (2011). Phytochemical characterisation for industrial use of pomegranate (*Punica granatum* L.) cultivars grown in Spain. J. Sci. Food Agric..

