# Supplementary material for: Phytochemical and antioxidant analysis of medicinal and food plants towards bioactive food and pharmaceutical resources
Source: Sci Rep. 2021 May 11;11:10041. doi: 10.1038/s41598-021-89437-4 (PMC8113553; doi:10.1038/s41598-021-89437-4)
Supplement: Supplementary file 1 — Supplementary Information 1. [file 41598_2021_89437_MOESM1_ESM.pdf]

Additional information

Abbreviation

$ABTS^{\bullet+}$: 2,2'-azino-bis (3-ethylbenzothiazoline-6-sulfonic acid) diammonium salt

CATE: catechin equivalents

$DPPH^{\bullet}$: 2,2-diphenyl-1-picrylhidrazyl radical

DW: dry weight

ECE: epicatechin equivalents

GAE: gallic acid equivalents

mM: m mol, millimole

RT: room temperature

TPTZ: 2,4,6-tris(2-pyridyl)-s-triazine

Trolox: (±)-6-hydroxy-2,5,7,8-tetramethylchromone-2-carboxylic acid
