# Supplementary material for: Phytochemical and antioxidant analysis of medicinal and food plants towards bioactive food and pharmaceutical resources
Source: Sci Rep. 2021 May 11;11:10041. doi: 10.1038/s41598-021-89437-4 (PMC8113553; doi:10.1038/s41598-021-89437-4)
Supplement: Supplementary file 2 — Supplementary Information 2. [file 41598_2021_89437_MOESM2_ESM.pdf]

Phytochemical and antioxidant analysis of medicinal and food plants towards bioactive food and pharmaceutical resources

Manyou Yu [1,*], Irene Gouvinhas [1], João Rocha [2] and Ana I.R.N.A Barros [1,3,*]

[1] *Centre for the Research and Technology of Agro-Environmental and Biological Sciences, CITAB, University de Trás-os-Montes e Alto Douro, UTAD, 5000-801, Vila Real, Portugal igouvinhas@utad.pt.*

[2] *Chemistry Centre -Vila Real, CQ-VR, UTAD, 5000-801, Vila Real, Portugal joaoffrocha@hotmail.com.*

[3] *Department of Chemistry, School of Life Sciences and Environment, UTAD, Quinta de Prados; 5001-801 Vila Real, Portugal*

* *Correspondence: jiangymy518@163.com; abarros@utad.pt.*

Supplementary Information:

Table S1 Information of studied plants in the study.

Figure S1 Chromatographic profile (at 280 nm) of a hydro-methanolic extract from pomegranate leaves. (1) *punicalin*; (2) ellagic acid derivative I; (3) *galloy glucose*; (4) *ellagitannin I (castalagin derivative)*; (5) *granatin B*; (6) ellagic acid derivative II (rhamnoside); (7) *ellagitannin II*; (8) *ellagitannin III*; (9) ellagic acid; (10) *ellagitannin IV*; (11) *ellagitannin V*; (12) apigenin glycoside II (glucoside); (13) luteolin glycoside VI (glucoside); (14) luteolin glycoside VII (glucoside). The compounds in italics were identified by literature; others by standards**.**

Figure S2 Chromatographic profile (at 280 nm) of a hydro-methanolic extract of sage (*Salvia officinalis* L.) leaves. (1) gallocatechin; (2) 2,3-hydroxybenzoic acid; (3) gallic acid derivative I; (4) catechin; (5) caffeic acid; (6) vanillic acid; (7) luteolin glycoside V; (8) apigenin glycoside III (rutinoside); (9) rosmarinic acid; (10) apigenin glycoside IV; (11) rosmanol; (12) epirosmanol; (13) *carnosol*; (14) *carnosic acid*. The compounds in italics were identified by literature; others by standards.

Figure S3 Chromatographic profile (at 280 nm) of a hydro-methanolic extract of rosemary (*Rosmarinus officinalis* L.) leaves. (1) gallocatechin; (2) gallic acid derivative I; (3) tyrosol; (4) catechin; (5) caffeic acid; (6) luteolin glycoside III; (7) luteolin glycoside V; (8) *eriodictyol-7-O-rutinoside*; (9) apigenin glycoside III (rutinoside); (10) rosmarinic acid; (11) luteolin-3-*O*-glucuronide;

(12) rosmanol; (13) epirosmanol; (14) *carnosol*; (15) *carnosic acid*. The compounds in italics were identified by literature; others by standards.

Figure S4 Chromatographic profile (at 280 nm) of a hydro-methanolic extract of rue (*Ruta graveolens* L.) leaves and young stems. (1) gallocatechin; (2) gallic acid derivative I; (3) neochlorogenic acid; (4) tyrosol; (5) coumaric acid; (6) chlorogenic acid; (7) vanillic acid; (8) rutin (quercetin-3-*O*-rutinoside); (9) quercetin glycoside I; (10) *psoralen*; (11) *8-methoxypsoralen*; (12) *5-methoxypsoralen*; (13) epicatechin gallate; (14) apigenin. The compounds in italics were identified by literature; others by standards.

Figure S5 Chromatographic profile (at 280 nm) of a hydro-methanolic extract of olive (*Olea europaea* L.) leaves. (1) gallocatechin; (2) 2,3-hydroxybenzoic acid; (3) gallic acid; (4) neochlorogenic acid; (5) tyrosol; (6) catechin; (7) chlorogenic acid; (8) vanillic acid; (9) *verbascoside*; (10) rutin (quercetin-3-*O*-rutinoside); (11) luteolin-7-O-glucoside; (12) apigenin glycoside I; (13) apigenin glycoside II (glucoside); (14) oleuropein derivative I; (15) oleuropein derivative II; (16) oleuropein; (17) oleuropein derivative III; (18) luteolin; (19) quercetin; (10) epicatechin gallate. The compounds in italics were identified by literature; others by standards.

Figure S6 Chromatographic profile (at 280 nm) of a hydro-methanolic extract of peppermint (*Mentha piperita* L.) leaves and young stems. (1) gallocatechin; (2) neochlorogenic acid; (3) catechin; (4) chlorogenic acid; (5) caffeic acid; (6) luteolin glycoside I; (7) *eriodictyol glycoside I (rutinoside)*; (8) rutin (quercetin-3-*O*-rutinoside); (9) luteolin glycoside II (rutinoside); (10) *eriodictyol glycoside II*; (11) luteolin glycoside IV (glucuronide); (12) *eriodictyol-7-O-rutinoside*; (13) rosmarinic acid; (14) apigenin glycoside IV; (15) luteolin; (16) epicatechin gallate. The compounds in italics were identified by literature; others by standards.

Figure S7 Chromatographic profile (at 280 nm) of a hydro-methanolic extract of parsley (*Petroselinum crispum* Mill.) leaves and young stems. (1) gallocatechin; (2) 2,3-hydroxybenzoic acid; (3) Gallic acid derivative I; (4) neochlorogenic acid; (5) catechin; (5) caffeic acid; (6) myricitin-3-O-glucoside; (7) epicatechin; (8) apigenin-7-O-apiosylglucoside (apiin); (9) *diosmetin glycoside*; (10) *diosmetin glycoside isomer*; (11) apigenin glycoside IV; (12) quercetin;(13) *naringenin*; (14) hesperidin; (15) epicatechin gallate. The compounds in italics were identified by literature; others by standards.

Table S1 Information of studied medicinal and food plants.

Scientific name (Common name)	Family	Parts used	Sampling date	Representative phenolics of methanolic leaf extracts in literature
Salvia officinalis L. (Sage)	Lamiaceae	Leaf	2018-05-09	hydroxybenzoic acid, gallic acid derivative, caffeic acid, vanillic acid, rosmarinic acid and its derivatives; catechin, gallocatechin, luteolin glycosides, apigenin glycosides
Rosmarinus officinalis L. (Rosemary)	Lamiaceae	Leaf	2018-05-09	gallic acid derivative, caffeic acid, rosmarinic acid and its derivatives; catechin, gallocatechin, luteolin glycosides, apigenin glycosides; tyrosol
Ruta graveolens L. (Rue)	Rutaceae	Leaf and young stem	2018-05-18	gallic acid derivative, neochlorogenic acid, coumaric acid, chlorogenic acid, vanillic acid; gallocatechin, rutin, quercetin glycosides, epicatechin gallate, apigenin; tyrosol
Olea europaea L. (Olive)	Oleaceae	Leaf	2018-05-18	hydroxybenzoic acid, gallic acid, neochlorogenic acid, chlorogenic acid, vanillic acid; gallocatechin, catechin, rutin, luteolin glycosides, apigenin glycosides, luteolin, quercetin, epicatechin gallate; tyrosol, verbascoside, oleuropein and its derivatives
Mentha piperita L. (peppermint)	Lamiaceae	Leaf and young stem	2018-06-25	neochlorogenic acid, chlorogenic acid, caffeic acid, rosmarinic acid; gallocatechin, catechin, luteolin glycosides, diosmetin glycosides, apigenin glycosides, luteolin, flavanone glycosides, epicatechin
Petroselinum crispum Mill. (Parsley)	Apiaceae	Leaf and young stem	2018-09-27	hydroxybenzoic acid, gallic acid derivative, neochlorogenic acid; gallocatechin, catechin, myricitin glycoside, epicatechin, apigenin glycosides, diosmetin glycosides, quercetin, epicatechin gallate
Punica granatum L. (Pomegranate)	Lythraceae	Leaf	2018-06-18	ellagic acid and its derivatives, gallic acid; luteolin glycosides, apigenin glycosides, kaempferol derivatives, gossypin, pentagalloyl glucose, quercetin, rutin; ellagitannin and its derivatives

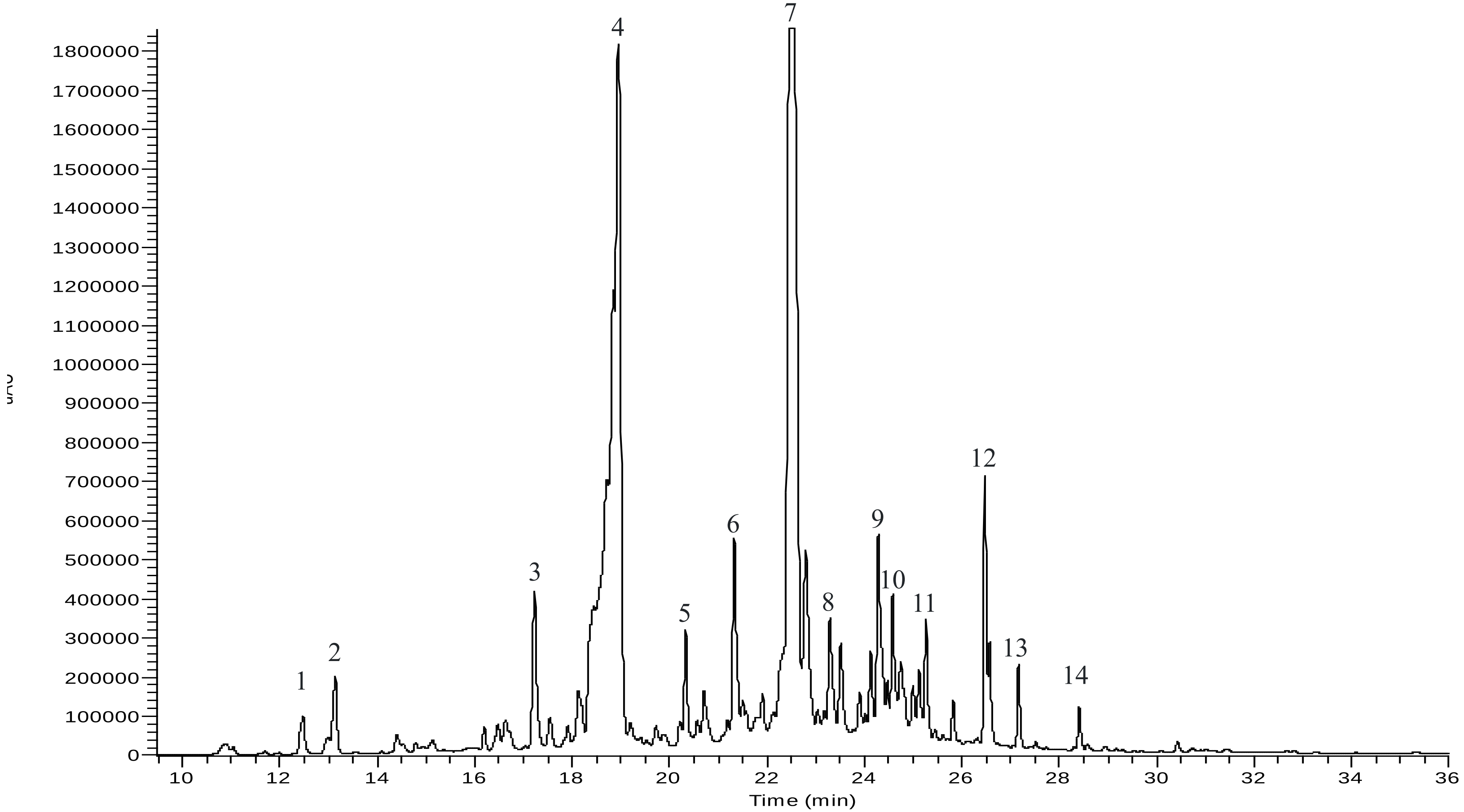

Figure S1 Chromatographic profile (at 280 nm) of a hydro-methanolic extract of pomegranate (*Punica granutum* L.) leaves. (1) *punicalin*; (2) ellagic acid derivative I; (3) *galloy glucose*; (4) *ellagitannin I (castalagin derivative)*; (5) *granatin B*; (6) ellagic acid derivative II (rhamnoside); (7) *ellagitannin II*; (8) *ellagitannin III*; (9) ellagic acid; (10) *ellagitannin IV*; (11) *ellagitannin V*; (12) apigenin glycoside II (glucoside); (13) luteolin glycoside VI (glucoside); (14) luteolin glycoside VII (glucoside). The compounds in italics were identified by literature; others by standards**.**

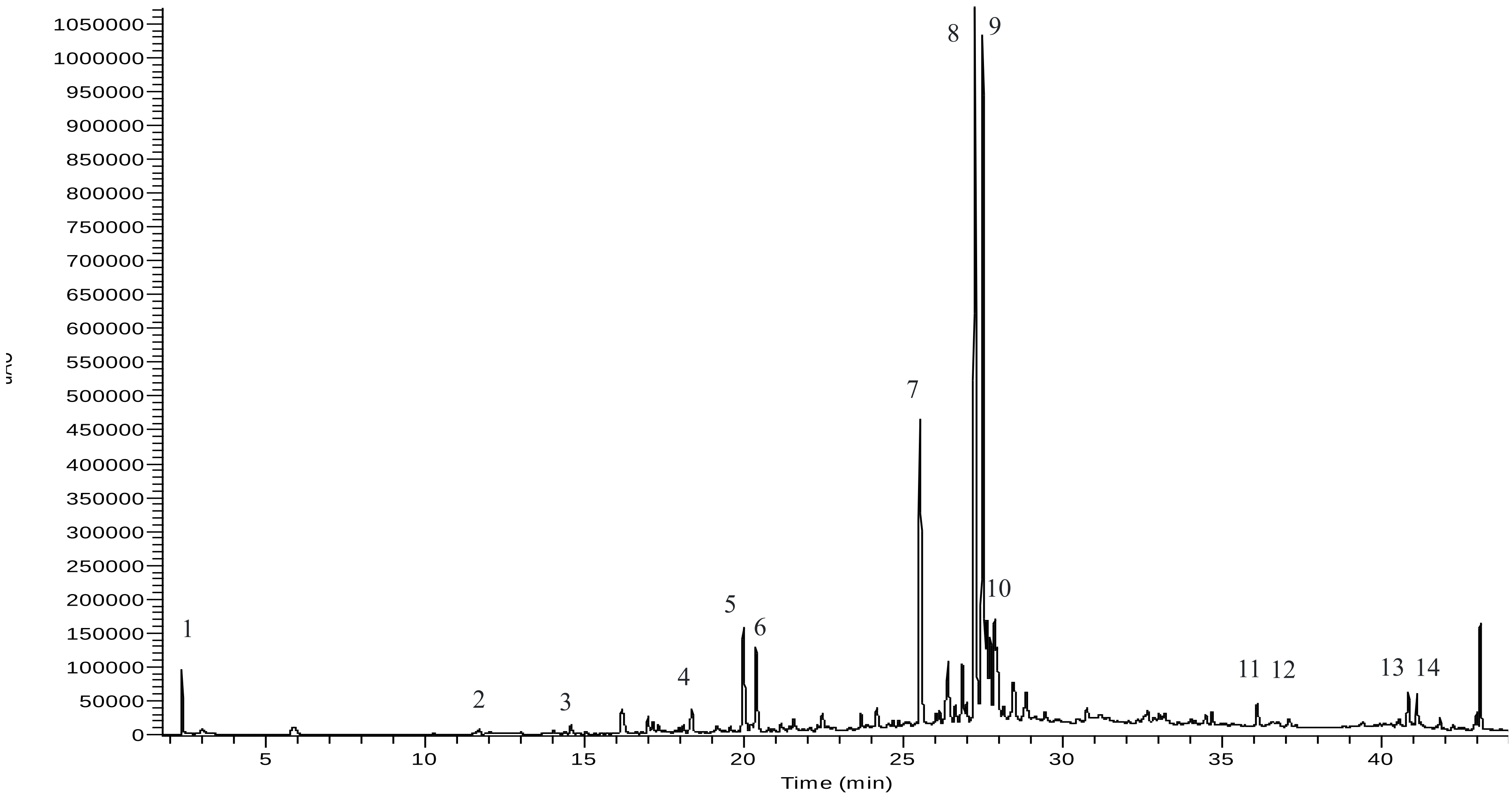

Figure S2 Chromatographic profile (at 280 nm) of a hydro-methanolic extract of sage (*Salvia officinalis* L.) leaves. (1) gallocatechin; (2) 2,3-hydroxybenzoic acid; (3) gallic acid derivative I; (4) catechin; (5) caffeic acid; (6) vanillic acid; (7) luteolin glycoside V; (8) apigenin glycoside III (rutinoside); (9) rosmarinic acid; (10) apigenin glycoside IV; (11) rosmanol; (12) epirosmanol; (13) *carnosol*; (14) *carnosic acid*. The compounds in italics were identified by literature; others by standards.

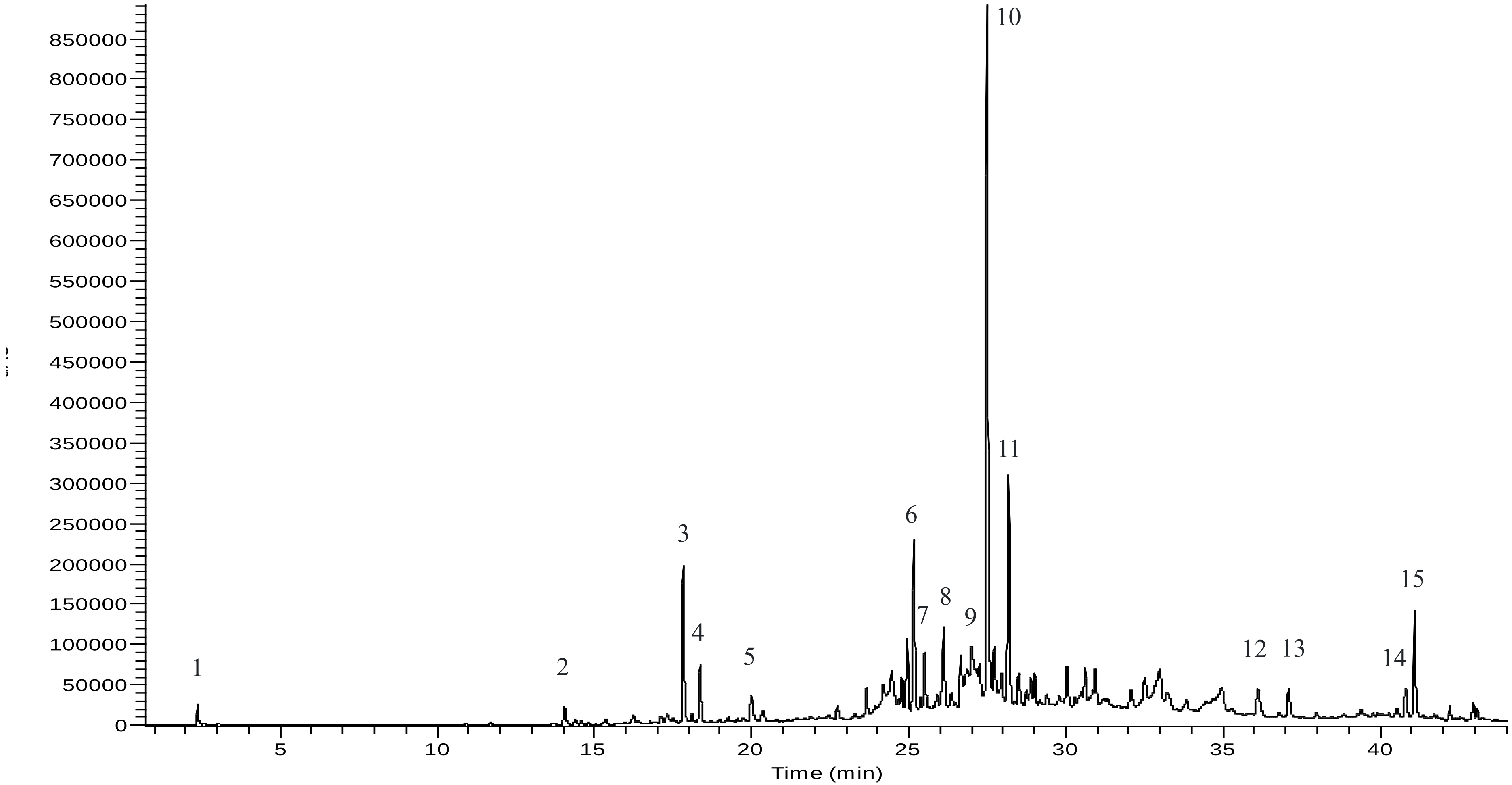

Figure S3 Chromatographic profile (at 280 nm) of a hydro-methanolic extract of rosemary (*Rosmarinus officinalis* L.) leaves. (1) gallocatechin; (2) gallic acid derivative I; (3) tyrosol; (4) catechin; (5) caffeic acid; (6) luteolin glycoside III; (7) luteolin glycoside V; (8) *eriodictyol-7-O-rutinoside*; (9) apigenin glycoside III (rutinoside); (10) rosmarinic acid; (11) luteolin-3-*O*-glucuronide; (12) rosmanol; (13) epirosmanol; (14) *carnosol*; (15) *carnosic acid*. The compounds in italics were identified by literature; others by standards.

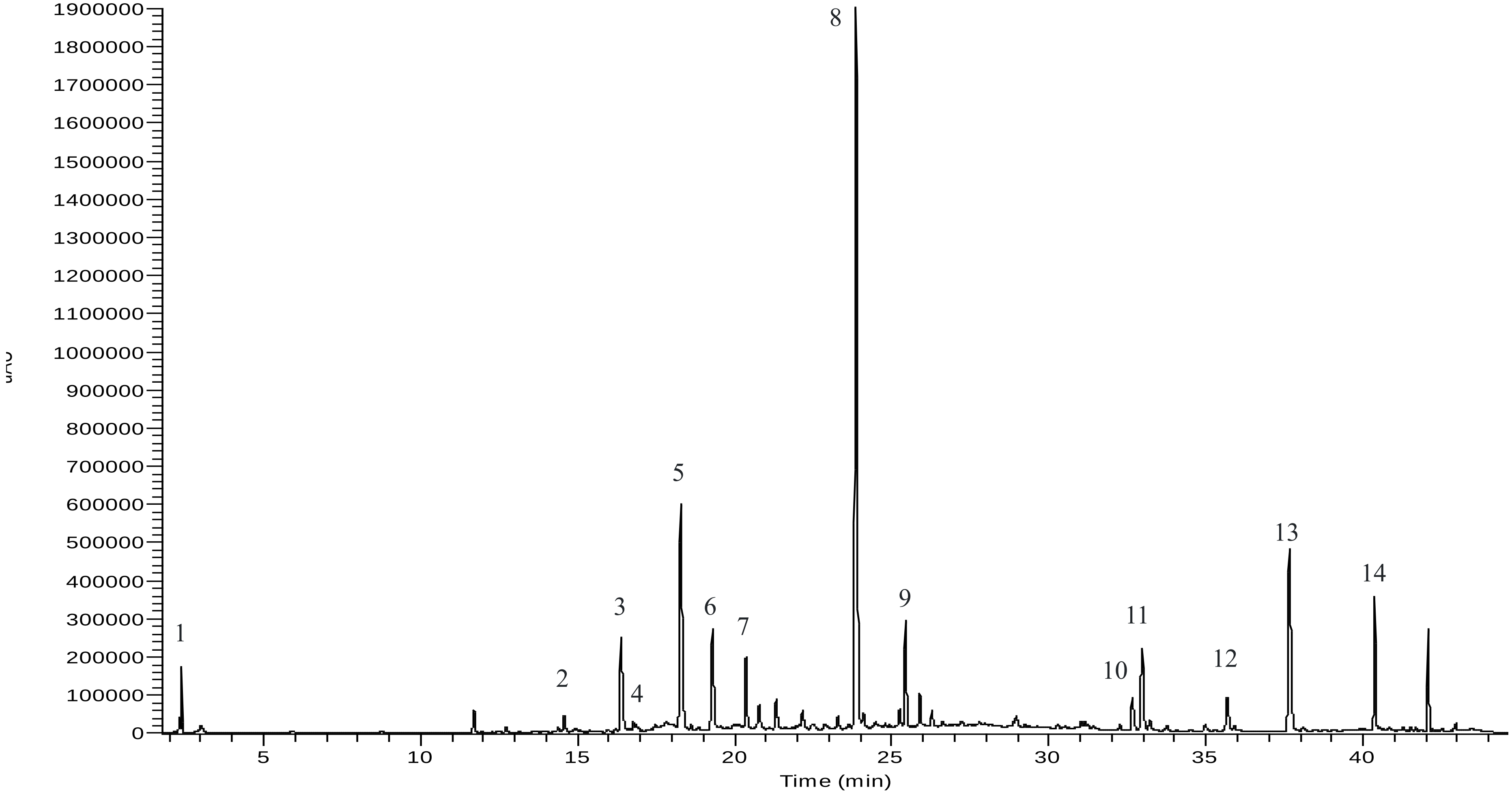

Figure S4 Chromatographic profile (at 280 nm) of a hydro-methanolic extract of rue (*Ruta graveolens* L.) leaves and young stems. (1) gallocatechin; (2) gallic acid derivative I; (3) neochlorogenic acid; (4) tyrosol; (5) coumaric acid; (6) chlorogenic acid; (7) vanillic acid; (8) rutin (quercetin-3-*O*-rutinoside); (9) quercetin glycoside I; (10) *psoralen*; (11) *8-methoxypsoralen*; (12) *5-methoxypsoralen*; (13) epicatechin gallate; (14) apigenin. The compounds in italics were identified by literature; others by standards.

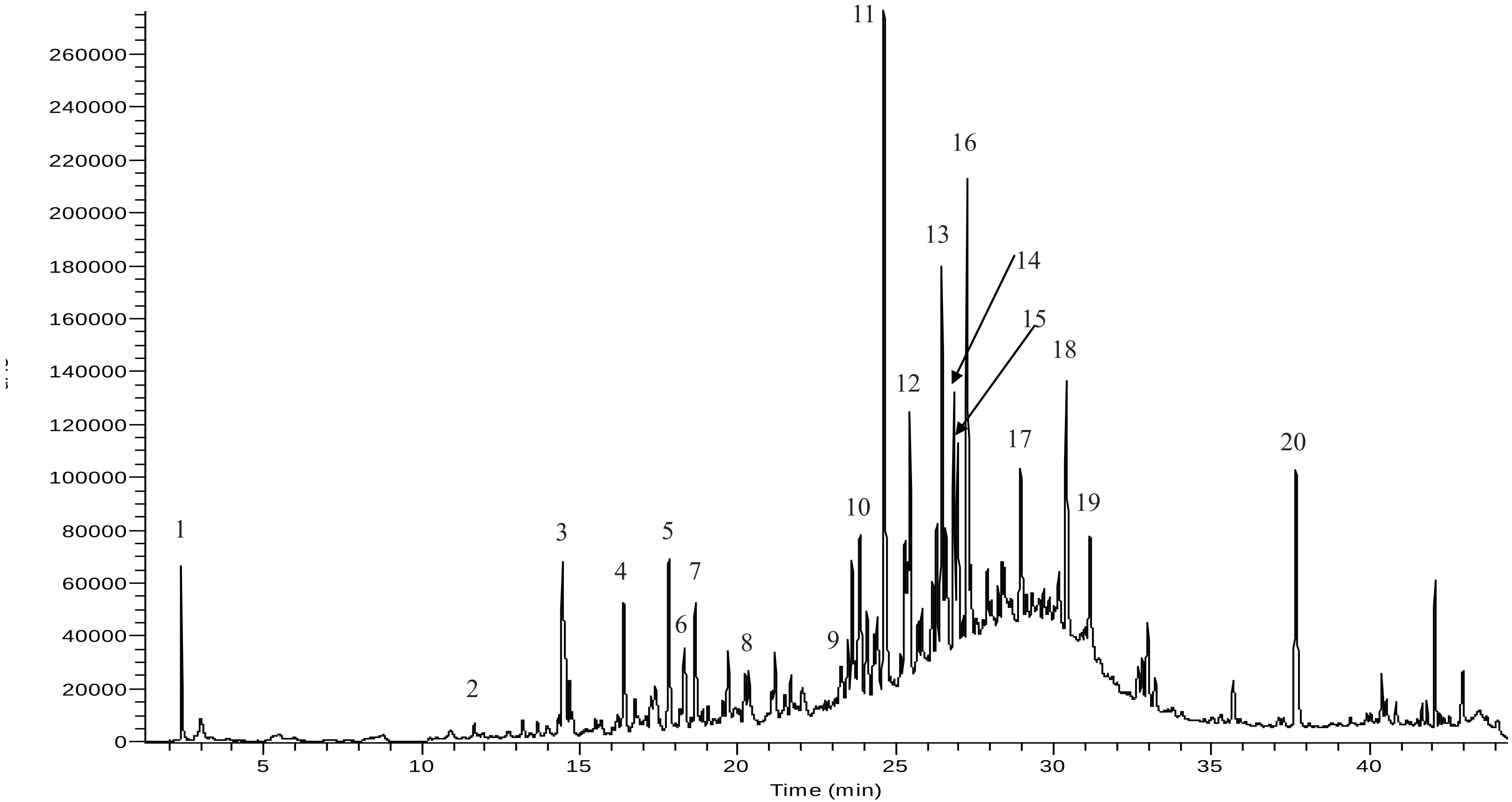

Figure S5 Chromatographic profile (at 280 nm) of a hydro-methanolic extract of olive (*Olea europaea* L.) leaves. (1) gallocatechin; (2) 2,3-hydroxybenzoic acid; (3) gallic acid; (4) neochlorogenic acid; (5) tyrosol; (6) catechin; (7) chlorogenic acid; (8) vanillic acid; (9) *verbascoside*; (10) rutin (quercetin-3-*O*-rutinoside); (11) luteolin-7-O-glucoside; (12) apigenin glycoside I; (13) apigenin glycoside II (glucoside); (14) oleuropein derivative I; (15) oleuropein derivative II; (16) oleuropein; (17) oleuropein derivative III; (18) luteolin; (19) quercetin; (10) epicatechin gallate. The compounds in italics were identified by literature; others by standards.

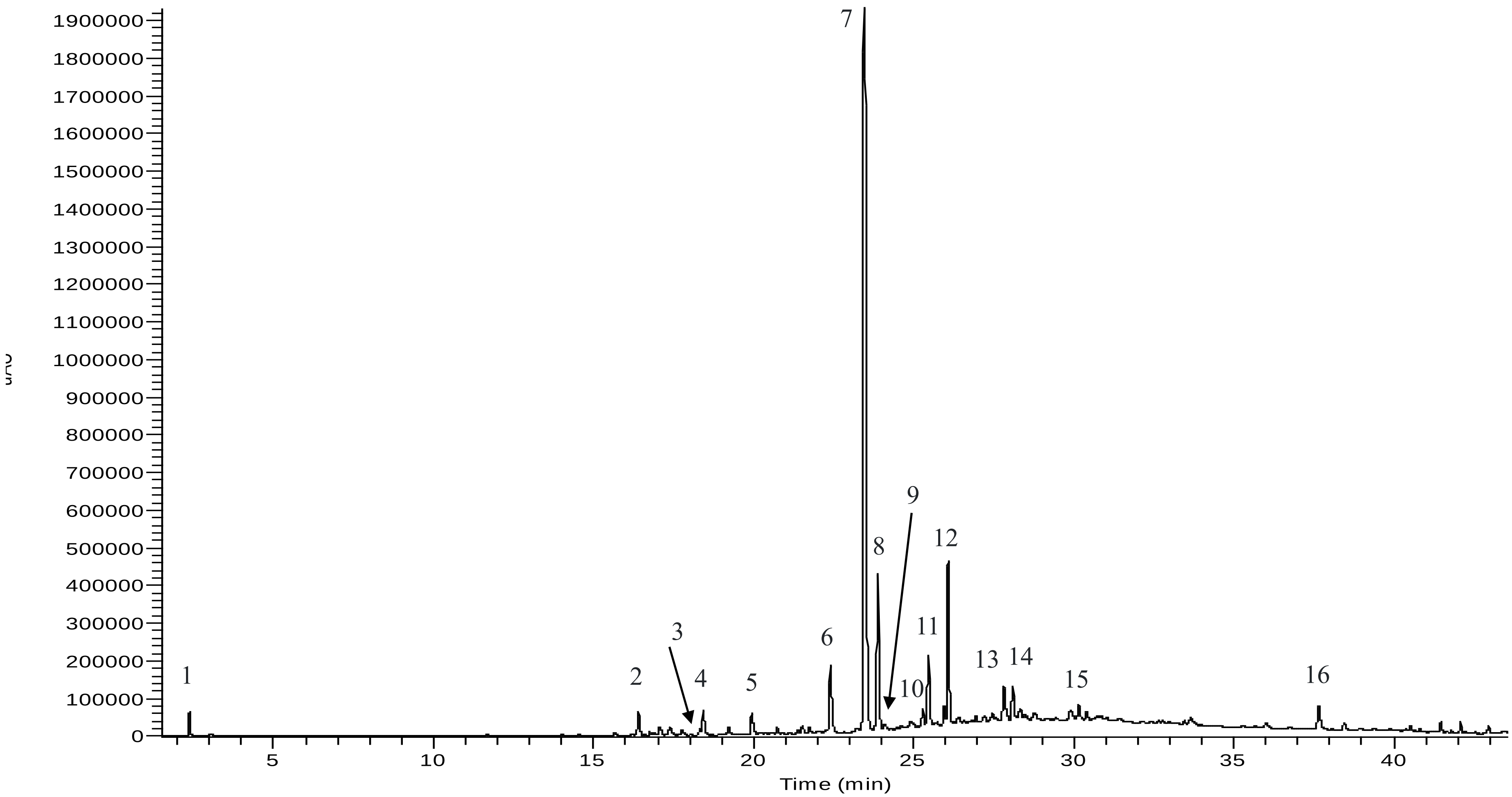

Figure S6 Chromatographic profile (at 280 nm) of a hydro-methanolic extract of peppermint (*Mentha piperita* L.) leaves and young stems. (1) gallocatechin; (2) neochlorogenic acid; (3) catechin; (4) chlorogenic acid; (5) caffeic acid; (6) luteolin glycoside I; (7) *eriodictyol glycoside I (rutinoside)*; (8) rutin (quercetin-3-*O*-rutinoside); (9) luteolin glycoside II (rutinoside); (10) *eriodictyol glycoside II*; (11) luteolin glycoside IV (glucuronide); (12) *eriodictyol-7-O-rutinoside*; (13) rosmarinic acid; (14) apigenin glycoside IV; (15) luteolin; (16) epicatechin gallate. The compounds in italics were identified by literature; others by standards.

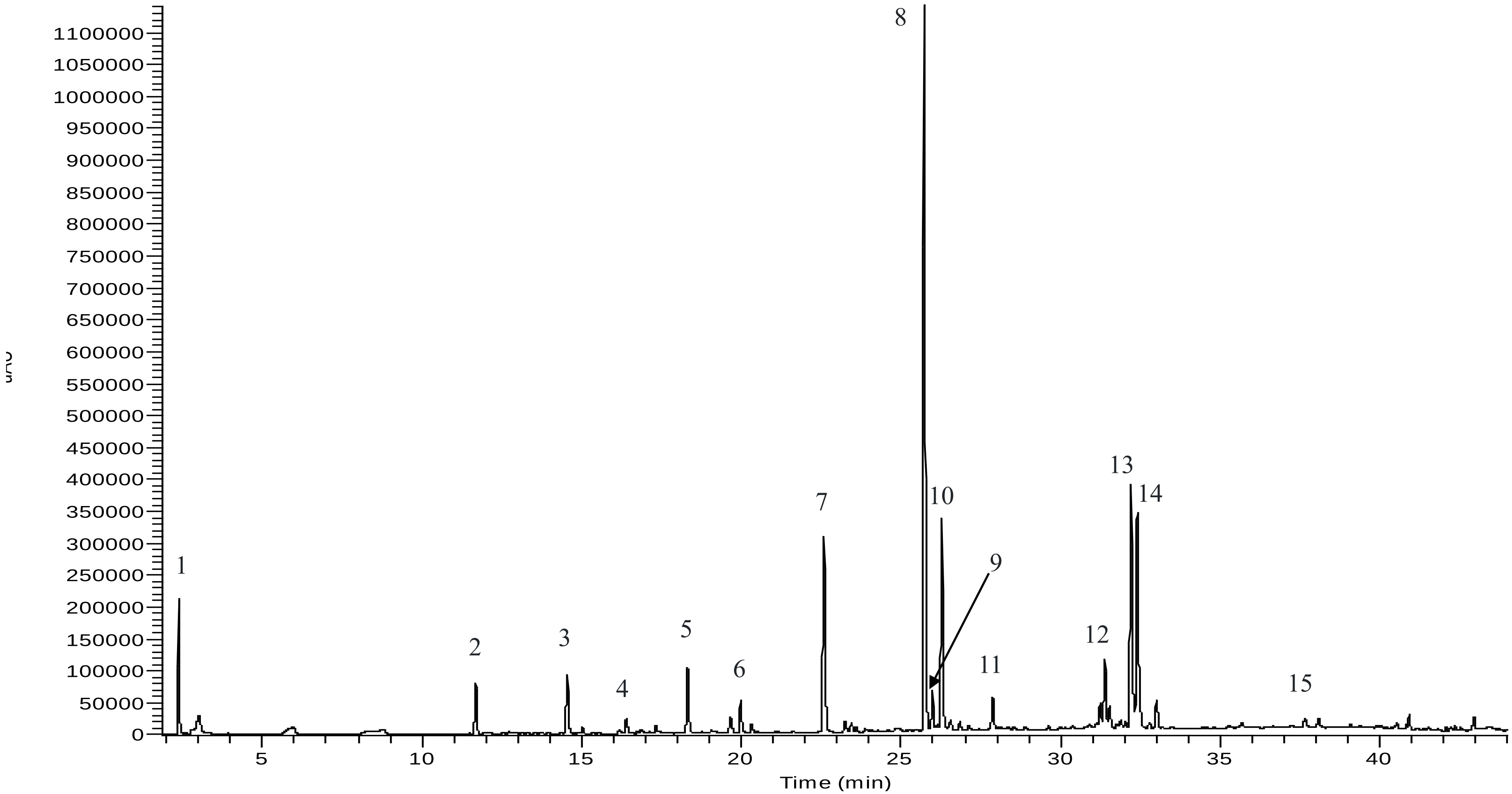

Figure S7 Chromatographic profile (at 280 nm) of a hydro-methanolic extract of parsley (*Petroselinum crispum* Mill.) leaves and young stems. (1) gallocatechin; (2) 2,3-hydroxybenzoic acid; (3) Gallic acid derivative I; (4) neochlorogenic acid; (5) catechin; (5) caffeic acid; (6) myricitin-3-O-glucoside; (7) epicatechin; (8) apigenin-7-O-apiosylglucoside (apiin); (9) *diosmetin glycoside*; (10) *diosmetin glycoside isomer*; (11) apigenin glycoside IV; (12) quercetin;(13) naringenin; (14) hesperidin; (15) epicatechin gallate. The compounds in italics were identified by literature; others by standards.
